# Multiple novel membrane proteins involve phthalate ester degradation in *Rhodococcus* sp. AH-ZY2

**DOI:** 10.1128/aem.02526-25

**Published:** 2026-04-29

**Authors:** Zhengyu Hou, Hejuan Pan, Shihan Wang, Yulin Li, Yang Yu, Pei Qiao, Haixia Wang, Weihong Zhong

**Affiliations:** 1 College of Biotechnology and Bioengineering, Zhejiang University of Technology 12624, Hangzhou, China; Danmarks Tekniske Universitet, Kgs. Lyngby, Denmark

**Keywords:** PAEs, MFS transporters, ABC transporters, substrate selectivity, transport mechanism, functional enhancement, molecular docking

## Abstract

Phthalate esters (PAEs) are ubiquitous in the environment, and their microbial metabolism is associated with transport proteins. In this study, multiple novel membrane proteins were identified, revealing their pivotal role in PAEs degradation. Based on whole-genome and transcriptomic analyses, approximately 20 potential PAEs transport membrane proteins were predicted to be involved in PAEs degradation by *Rhodococcus* sp. AH-ZY2. Among them, four protein genes (*0620, 3572, 4497,* and *5299*) exhibited increased transcription levels (Log_2_F(c)>2.8) in response to di-n-octyl-phthalate (DnOP) as the sole carbon source, instead of fructose, were selected for functional verification. Gene knockout and complementation experiments showed that these four transport protein genes, belonging to the MFS family (*0620*, *3572*, and *5299*) and the ABC family (4497), respectively, exhibited the function to transport multiple PAEs into cells. Specifically, membrane protein 5299 could transport the most diverse kinds of PAEs. Molecular docking results also showed that Ser and Arg play a key role in the PAEs transport process of membrane protein 5299, and the PAEs-binding pocket size of 5299 in the transition region was larger than that of 0620, 3572, and 4497. Then, the expression of the genes encoding 0620, 3572, 5299, and 4497 was enhanced after they were cloned into the plasmid pNV18 and transformed into the strain AH-ZY2. The enhancement of 5299 expression exhibited a better improvement in PAEs degradation than the other three genes. This study provides four novel transport proteins and a new strategy for effective bioremediation of PAEs via enhanced membrane transport.

**IMPORTANCE:**

Phthalate esters (PAEs) are widely present in the environment, with carcinogenic, teratogenic, and mutagenic toxicity to the human body. The efficient microbial degradation of PAEs is urgent for eco-friendly bioremediation. In addition to PAE esterases, membrane proteins for PAE transport are also important for the microbial degradation of PAEs. However, few experimental reports on the membrane proteins involved in PAE transport, and specifically no studies regarding their underlying transport mechanisms, have been published. Therefore, investigation of the PAE transport mechanisms is crucial for understanding how PAEs enter and exit cells, and it contributes to identifying the rate-limiting steps in PAE degradation. It is conducive to revealing the role of membrane proteins in PAEs degradation, for improving PAE degradation efficiency via membrane protein engineering, or endowing other chassis cells with PAEs degradation capability via constructing membrane protein-esterase co-expressing strains.

## INTRODUCTION

Phthalate esters (PAEs) in plastics are synthetic organic compounds that are classified as endocrine disruptors. They can easily volatilize into the environment, leading to pollution (1–4). Mixed contamination of PAEs has been identified in various environmental matrices, including food, water, soil, and air (5–8). An increasing number of microbial strains capable of degrading PAEs have been isolated from polluted environments. However, there are few literature reports on the transport mechanisms of PAEs into cells (9–11). During bacterial PAE degradation, passive diffusion may damage cellular integrity and function; consequently, bacteria may primarily rely on membrane protein-mediated transport for PAE assimilation (12–14). So far, only one of the PAE membrane proteins, an ATP-binding-cassette transporter PatDABC, has been proven to transport PAEs across the cytomembrane in the gram-positive bacterium *Rhodococcus jostii* RHA1 (10). However, an increasing number of membrane proteins have been proven to transport various aromatic compounds for bacterial degradation (Table S1). For instance, a novel outer membrane protein FadL was found, as the rate-limiting step for bacterial degradation of PAH (15), while AltL is primarily responsible for transporting long-chain n-alkanes and fatty acids (16). Both MdfA and TajR from *E. coli* can transport chloramphenicol into cells (17, 18). These findings collectively demonstrate that membrane proteins play a crucial role in the microbial degradation of organic pollutants, possibly including PAEs.

Membrane proteins play a vital role in the life activities of bacteria (19–21). Gram-negative bacteria possess an additional outer membrane compared to gram-positive bacteria. Consequently, a greater variety of transport proteins, such as OmpW (outer membrane protein W), FadL (fatty acid transport protein), and TBDT (TonB-dependent transporter), are found in the outer membrane (15, 22–24). MFS (major facilitator superfamily) and ABC (ATP-binding cassette) transporters are located on the inner membrane of cells (25, 26).

MFS transporters typically consist of 12–14 transmembrane helical domains, which create a central channel or binding pocket for the accommodation and transport of substrate molecules (25). The MFS transport mechanism is complex. Quistgaard et al. propose an updated model for the conformational cycle of MFS transporters, the “clamp-and-switch model” (27). MFS transporters may be associated with the degradation of aromatic compounds (17, 18, 28–37). For instance, the Vank protein in *Pseudomonas putida* KT2440 belongs to the aromatic acid/hydrocarbon symporter (AAHS) of the MFS family and can transport protocatechuic acid and vanillate (37). The Hcnk protein of *Pseudomonas putida* KT2440 belongs to the AAHS of the MFS family and is capable of transporting 4-Coumarate and Ferulate (37). ABC transporters usually consist of transmembrane domains (TMDs), nucleotide-binding domains (NBDs), and substrate-binding proteins (SBPs). Their transport processes are strictly coupled with ATP hydrolysis (38). Hara et al. proved that ABC transporters are related to the transport of mono-phthalate esters (MAPs) through omics analysis (10). The ABC transporter system DmpXWV found in marine bacteria can specifically transport dimethylsulfoniopropionate (DMSP) (39).

In our previous study, three effective PAE-degrading strains, *Glutamicibacter* sp. ZJUTW, *Gordonia* sp. GZ-YC7, and *Rhodococcus* sp. AH-ZY2, were isolated and identified (40–42). AH-ZY2 has a broader substrate spectrum and higher degradation efficiency than ZJUTW and GZ-YC7 (Table S2). This phenomenon may result from the difference in their membrane proteins and esterases of PAEs (Table S3).

The transcriptomic results revealed 31 upregulated genes (log_2_F(c) > 0.5) in ZJUTW responded to dibutyl phthalate (DBP) belonged to the MFS membrane protein genes; 12 upregulated genes (log_2_F(c) > 0.5) in GZ-YC7 responded to Bis(2-ethylhexyl) phthalate (DEHP) belonged to the MFS membrane protein genes; 27 upregulated genes (with log_2_F(c) > 0.5) in AH-ZY2 responded to DnOP belonged to the MFS membrane protein genes (Table S4). Although there exist more upregulated MFS transporters in ZJUTW than in AH-ZY2, ZJUTW can only degrade short-chain PAEs. Based on the combined results of membrane protein and degradation characteristics, AH-ZY2 is more worthy of further research than ZJUTW and GZ-YC7. Therefore, this study focuses on the membrane proteins only in AH-ZY2.

In addition, it was hypothesized that the substrate selectivity and high efficiency for PAEs degradation in AH-ZY2 may relate to both its multiple PAEs esterases and membrane transportation proteins. Thus, this study aims to elucidate the identity and functional mechanisms of key membrane transporters. We also hypothesize that specific MFS and ABC transporters concertedly mediate efficient PAE uptake, and that loss of their function will significantly impair uptake and degradation, suggesting transport as a potential rate-limiting step in PAE biodegradation. This work provides the first mechanistic insight into PAE transmembrane transport, offering a critical theoretical basis for enhancing bioremediation by regulating microbial substrate uptake efficiency.

## RESULTS

### Mining of membrane proteins for PAEs transport in *Rhodococcus* sp. AH-ZY2

Gene coverage analysis based on RSeQC revealed that the sequencing reads were uniformly distributed across gene transcripts, with no distinct peaks observed at either the 5′ or 3′ ends. This indicates the absence of sequence positional bias, confirming that the data are uniform and reliable and suitable for subsequent quantitative expression analysis (Fig. S1).

In our previous study, 27 upregulated MFS genes have been mined in response to DnOP (as a representative of long-chain PAEs). However, the transporter genes in response to other kinds of PAEs still require clarification. Thus, the membrane protein genes in response to DBP (as a representative of short-chain PAEs) and mixed PAEs (DMP, dimethyl phthalate; DEP, diethyl phthalate; DPrP, dipropyl phthalate; DBP, dibutyl phthalate; BBP, benzyl butyl phthalate; DEHP, bis(2-ethylhexyl) phthalate; DnOP, di-n-octyl-phthalate; DiNP, diisononyl phthalate) were also mined via RNA-seq technology in this study. The results showed that the numbers of highly upregulated (Log_₂_F(c) > 2) MFS transporter genes in response to DBP, DnOP, and mixed PAEs were 2, 4, and 5, respectively (Table 1), while the numbers of highly upregulated (Log_₂_F(c) > 2) ABC protein gene clusters were 2, 6, and 2, respectively (Table 2). AH-ZY2 exhibited the highest efficiency in degrading DnOP. Therefore, the transporter protein genes in response to DnOP were selected for in-depth study. Among the above-mentioned proteins genes, the top three upregulated MFS family proteins genes in response to DnOP (**5299**, Log_2_F(c) = 5.194; **0620**, Log_2_F(c) = 3.707; and **3572**, Log_2_F(c) = 2.871) and one ABC family protein gene (**4497**, Log_2_F(c) = 3.282) from an all-upregulated cluster were selected for in-depth study (Table 2).

**TABLE 1:** Highly upregulated (Log_2_F(c) > 2) MFS membrane proteins in AH-ZY2 strain response to different PAEs^*a*^

PAE	Gene number	Log_2_F(c)	Type	*P*-value	Function
DBP	*2841*	3.043	Up	***	MFS transporter
	*3107*	2.972	Up	***	MFS transporter
DnOP	***5299***	**5.194**	**Up**	***	**MFS transporter**
	***0620***	**3.707**	**Up**	***	**MFS transporter**
	***3572***	**2.871**	**Up**	***	**MFS transporter**
	*2875*	2.335	Up	***	MFS transporter
Mixed PAEs	*5178*	5.141	Up	***	MFS transporter
	*2138*	4.325	Up	***	MFS transporter
	*2841*	2.517	Up	***	MFS transporter
	*2414*	2.282	Up	***	MFS transporter
	*2931*	2.156	Up	***	MFS transporter

^*a*^ Mixed PAEs (DMP, DEP, DPrP, DBP, BBP, DEHP, DnOP, and DiNP). ***, *P* < 0.001; **, 0.001 < *P* < 0.01; *, 0.01 < *P* < 0.05; ns, *P* > 0.05. Bold values represent a membrane protein that is the focus of research.

**TABLE 2:** Highly upregulated (Log_2_F(c) > 2) ABC membrane proteins in AH-ZY2 strain response to different PAEs^*a*^

PAE	Gene cluster	Gene number	Log_2_F(c)	Type	*P*-value	Function
DBP	1	*3753*	2.463	Up	***	ABC substrate-binding protein
		*3754*	1.583	Up	***	ABC transporter permease subunit
		*3755*	2.171	Up	***	ABC transporter permease
		*3756*	2.183	Up	***	ABC ATP-binding protein
	2	*3994*	1.999	Up	***	ABC substrate-binding protein
		*3995*	3.224	Up	***	ABC ATP-binding protein
		*3996*	1.025	Up	***	ABC transporter permease protein
DnOP	1	***4497***	**3.282**	**Up**	***	**ABC substrate-binding protein**
		***4498***	**3.625**	**Up**	***	**ABC ATP-binding protein**
		***4499***	**5.038**	**Up**	***	**ABC transporter permease**
	2	*1589*	1.381	Up	**	ABC transporter permease
		*1590*	0.526	Up	ns	ABC transporter permease
		*1591*	0.521	Up	ns	ABC substrate-binding protein
		*1592*	2.531	Up	**	ABC substrate-binding protein
		*1593*	2.624	Up	***	ABC substrate-binding protein
	3	*1926*	3.144	Up	***	ABC substrate-binding protein
		*1927*	4.048	Up	***	ABC transporter permease
		*1928*	0.778	Up	***	ABC transporter permease
	4	*2003*	4.503	Up	***	ABC transporter permease protein
		*2004*	2.593	Up	***	ABC transporter permease
		*2005*	1.215	Up	**	ABC substrate-binding protein
	5	*2438*	2.193	Up	***	ABC transporter permease
		*2439*	2.059	Up	***	ABC transporter permease
		*2440*	1.863	Up	***	ABC substrate-binding protein
		*2441*	0.534	Up	***	ABC ATP-binding protein
	6	*3994*	2.672	Up	***	ABC substrate-binding protein
		*3995*	2.922	Up	***	ABC ATP-binding protein
		*3996*	2.260	Up	***	ABC transporter permease protein
Mixed PAEs	1	*4217*	−0.173	Down	***	ABC ATP-binding protein
		*4218*	1.050	Up	***	ABC transporter permease
		*4219*	2.447	Up	***	ABC transporter permease
		*4220*	2.798	Up	***	ABC substrate-binding protein
	2	*4312*	3.323	Up	***	ABC substrate-binding protein
		*4313*	4.284	Up	***	ABC transporter permease
		*4314*	1.602	Up	***	ABC transporter permease
		*4315*	0.997	Up	***	ABC ATP-binding protein

^*a*^ Mixed PAEs (DMP, DEP, DPrP, DBP, BBP, DEHP, DnOP, and DiNP). ***, *P* < 0.001; **, 0.001 < *P* < 0.01; *, 0.01 < *P* < 0.05; ns, *P* > 0.05. Bold values represent a membrane protein that is the focus of research.

**Fig 1:**
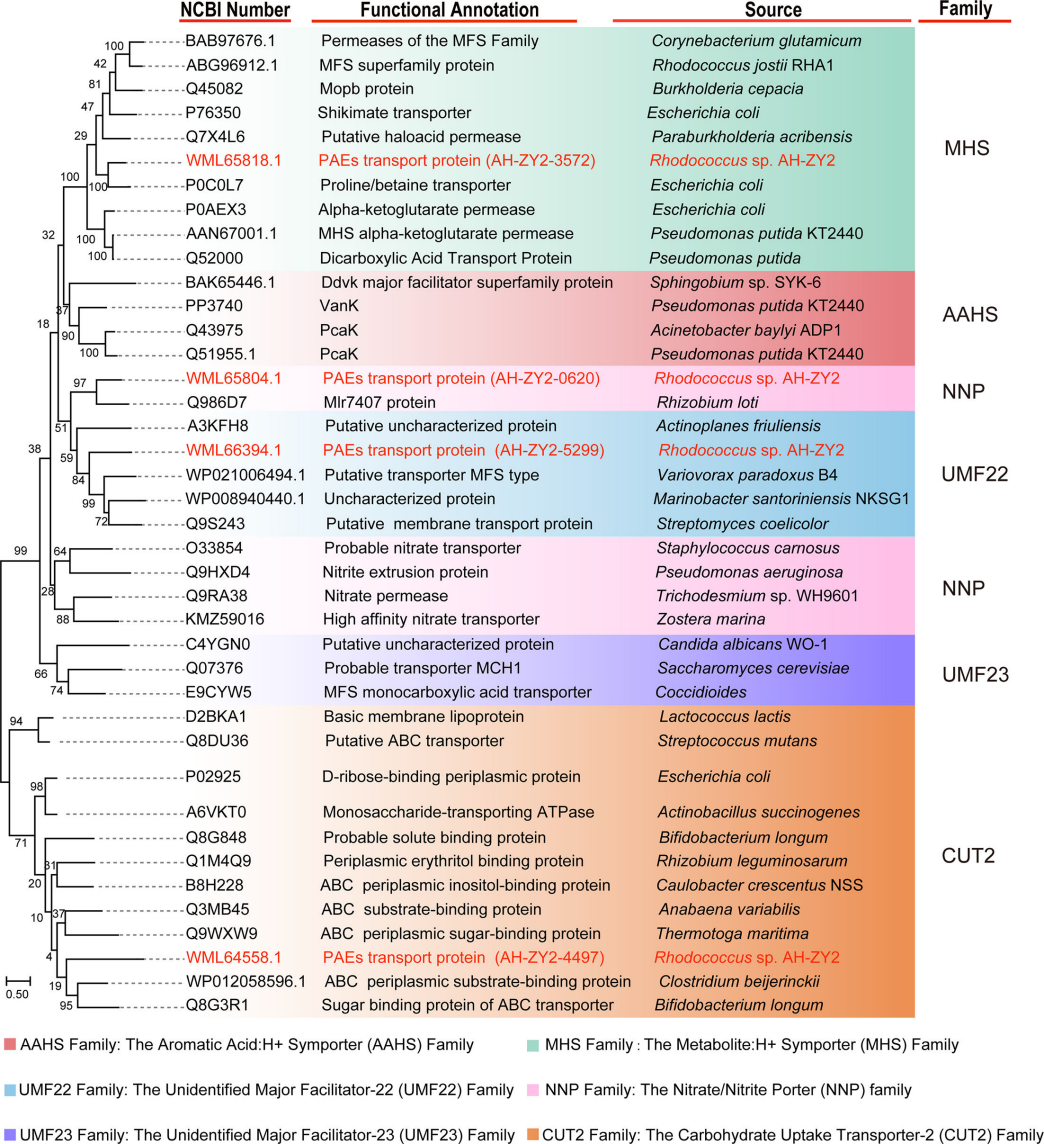
Phylogenetic tree of transporters in strain AH-ZY2 belonging to MFS and ABC transporter families. The MHS, AAHS, UMF22, UMF23, and NNP belong to MFS families. The CUT2 belongs to the ABC transporter family. Since the ABC transporter family consists of three proteins, and 4497 belongs to the substrate-binding protein, the substrate-binding proteins of ABC transporters were selected for constructing the phylogenetic tree. Red indicates the transporters studied in this article.

After NCBI and TCDB blast comparison, membrane proteins 5299, 4497, 0620, and 3572 were similar to uncharacterized major facilitator-22 (UMF22) family (with highest similarity of 21.7% to an uncharacterized protein), the carbohydrate uptake transporter-2 (CUT2) family (with highest similarity of 17.4% to a periplasmic substrate-binding protein), the nitrate/nitrite Porter (NNP) family (with highest similarity of 28.0% to Mlr7407), and the metabolite, H+ ymporter (MHS) family (with highest similarity of 37.0% to proline/betaine transporters), respectively. The phylogenetic tree also showed that membrane protein 5299 belongs to the UMF22 family, membrane protein 4497 belongs to the CUT2 family, 0620 belongs to the NNP family, and 3572 belongs to the MHS family (Fig. 1). This result is consistent with those obtained from alignments using TCDB and NCBI databases. The phylogenetic tree and sequence alignment results confirm the accuracy of the family classification of membrane proteins 5299, 4497, 0620, and 3572.

### Effect of membrane protein gene knockout on cell growth and PAE degradation efficiency

**Fig 2:**
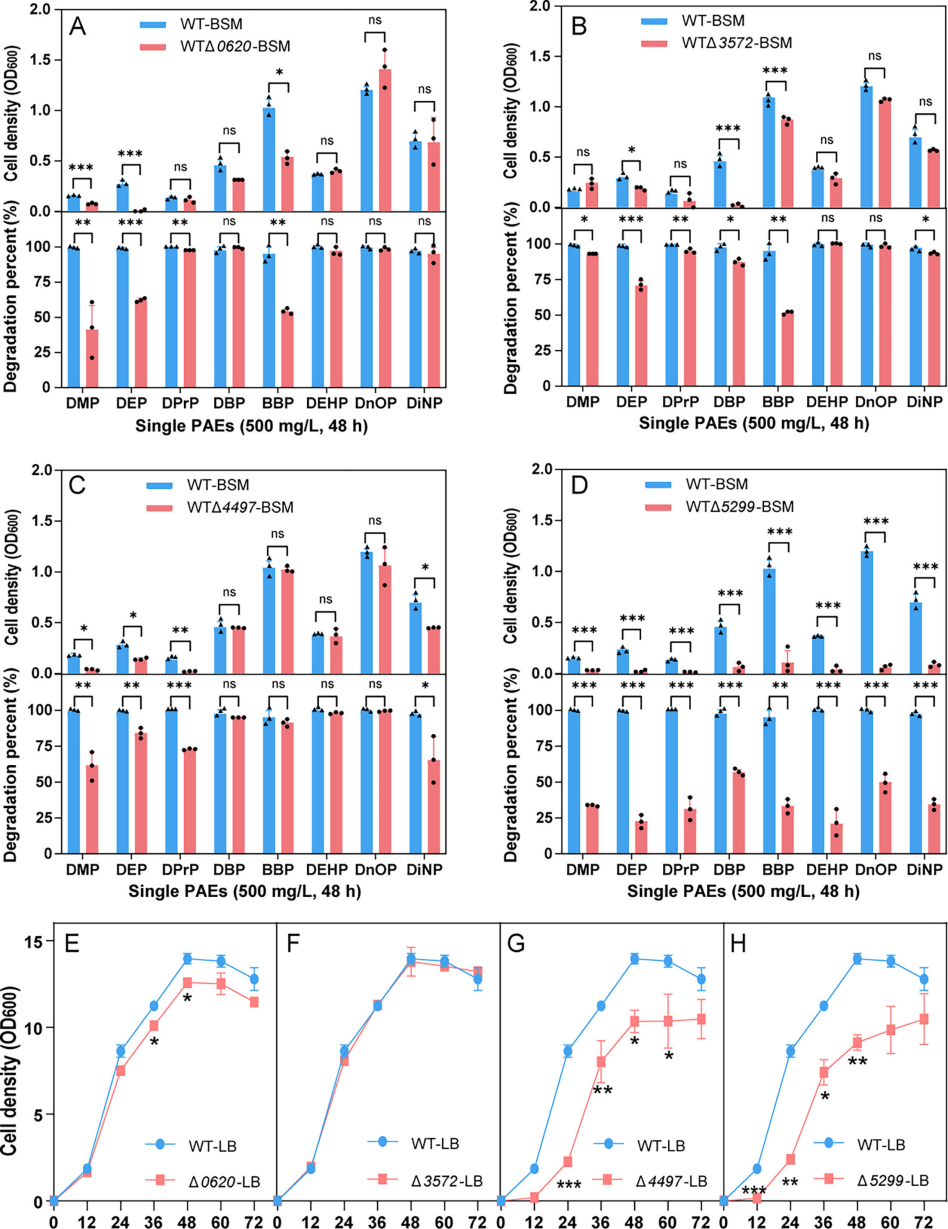
Effect of the membrane protein genes knockout on the cell growth and PAEs degradation efficiency in BSM and LB containing 500 mg/L of eight different single PAEs. Δ*0620* (**A and E**), Δ*3572* (**B and F**), Δ*4497* (**C and G**), and Δ*5299* (**D and H**), respectively, in BSM (48 h) and LB medium (72 h) containing 500 mg/L single PAEs. Bars represent mean ± SD from three independent experiments (*n* = 3). ***, *P* < 0.001; **, 0.001 < *P* < 0.01; *, 0.01 < *P* < 0.05; ns, *P* > 0.05.

Membrane protein genes *0620*, *3572*, *4497*, and *5299* were successfully knocked out, respectively (Fig. S2 to S5), and then the cell growth and degradation of PAEs by resultant knockout bacteria were evaluated. In basic inorganic salt medium (BSM), transporter gene 0620 knockout impaired the growth on DMP, DEP, DBP, and BBP, whereas no influence was detected on DPrP, DEHP, DnOP, or DiNP (Fig. 2A). Transporter gene 3572 knockout affected the degradation of DEP, DBP, and BBP, without altering that of DMP, DPrP, DEHP, DnOP, or DiNP (Fig. 2B). Transporter gene 4497 knockout significantly reduced growth on DMP, DEP, and DiNP (Fig. 2C). Transporter gene 5299 knockout caused significant growth defects on all eight kinds of PAEs, showing its broad substrate response (Fig. 2D). Meanwhile, the degradation rate of eight PAEs by Δ*5299* was significantly lower than WT in basic inorganic salt medium (BSM) (Fig. 2D). However, during the exponential growth phase (12–48 h) in lysogeny broth (LB) medium, the average growth rates of WT, Δ*3572*, Δ*0620*, Δ*4497*, and Δ*5299* were 0.50 h⁻¹, 0.49 h⁻¹, 0.45 h⁻¹, 0.42 h⁻¹, and 0.37 h⁻¹, respectively (Fig. 2E through H). Thus, no significant growth difference was detected between Δ*3572* and WT, indicating protein 3572 is not related to normal metabolism. Whereas Δ*0620*, Δ*4497*, and Δ*5299* grew worse than WT, suggesting proteins 0620, 4497, and 5299 are related to normal metabolism of the strain AH-ZY2 (Fig. 2).

**Fig 3:**
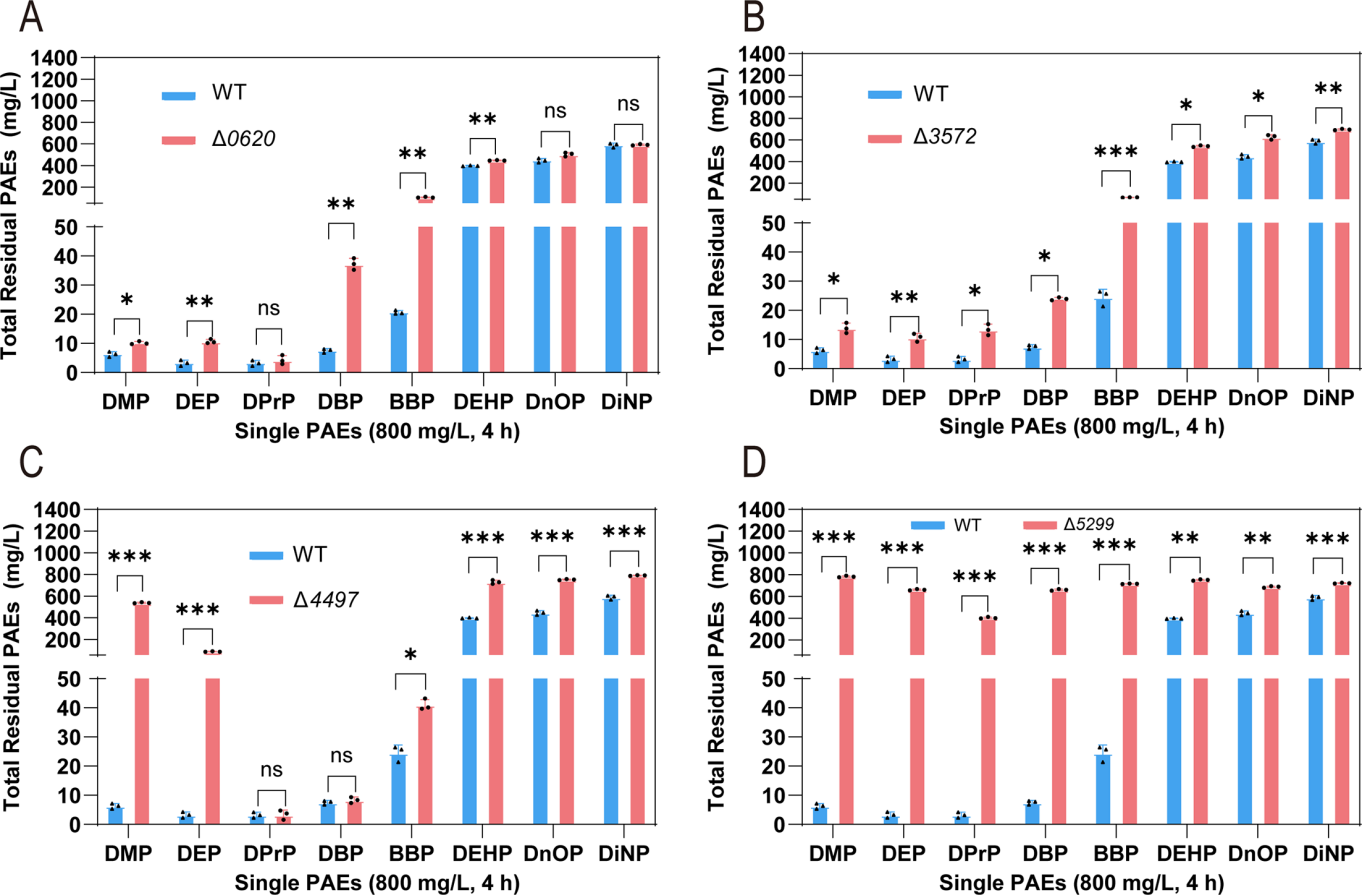
Effect of the membrane protein gene knockout on the degradation efficiency of eight different individual PAEs (800 mg/L) after 4 h whole-cell degradation. Total residual PAEs comparison between WT and Δ*0620* (**A**), Δ*3572* (**B**), Δ*4497* (**C**), and Δ*5299* (**D**). Bars represent mean ± SD from three independent experiments (*n* = 3). ***, *P* < 0.001; **, 0.001 < *P* < 0.01; *, 0.01 < *P* < 0.05; ns, *P* > 0.05.

When the eight single PAEs were degraded by the whole cells of the WT and knockout strains, the change of total residual PAEs (intracellular + extracellular) confirmed that 0620, 3572, 4497, and 5299 could affect the degradation of multi-PAEs (Fig. 3). Δ*0620* showed impaired degradation of DMP, DEP, DBP, BBP, and DEHP, with the degradation rate of BBP decreasing by 10.59% (Fig. 3A). Δ*3572* exhibited a decrease in the degradation rate of all eight PAEs, with a more pronounced reduction observed for long-chain PAEs. The degradation rates of DEHP, DnOP, and DiNP decreased by 18.08%, 22.22%, and 13.97%, respectively (Fig. 3B). Δ*4497* showed reduced degradation rates for DMP, DEP, BBP, DEHP, DnOP, and DiNP, by 66.45%, 10.59%, 2.64%, 41.50%, 38.07%, and 45.25%, respectively (Fig. 3C). Δ*5299* showed decreased degradation rates for all eight PAEs (DMP, DEP, DPrP, DBP, BBP, DEHP, DnOP, and DiNP) by 97.08%, 82.35%, 49.98%, 81.57%, 86.84%, 44.04%, 29.95%, and 17.03%, respectively (Fig. 3D).

### Change of extracellular and intracellular PAEs after transporter knockout

Extracellular and intracellular PAEs concentrations were measured in the knockout strains and the WT. Compared with the WT, the knockout strains exhibited significantly elevated extracellular levels of PAEs. Specifically, the extracellular levels of DMP, DEP, and BBP, in both Δ*0620* and Δ*3572*; DMP, DEP, DEHP, DnOP, and DiNP in Δ*4497*; all PAEs in Δ*5299* were significantly increased (Fig. S6A, C, E, and G). These results suggest that proteins 0620, 3572, 4497, and 5299 mediate the transport of specific PAEs into cells. No significant changes in intracellular PAEs concentrations were detected in the membrane transporter knockout strains (Fig. S6B, D, F and H). Only the intracellular BBP concentration of the Δ*5299* strain appeared to be increased (Fig. S6H). This may reflect not the actual situation because the highest level of attached BBP among all samples may result in its release into the supernatant of the mixture after ultrasonication, thereby increasing the apparent intracellular concentration of BBP.

### Restoration of growth on PAEs by membrane protein in plasmid-complemented strains

Complementation plasmids pNV18-*0620*, pNV18-*3572*, pNV18-*4497*, and pNV18-*5299* were constructed and introduced into their respective knockout strains. After 48 h cultivation, compared with the strain Δ*0620*, pNV18-*0620* restored the growth of the knockout strain on DMP, DEP, and DBP, confirming that protein 0620 specifically transports these PAEs (Fig. S7A). Compared with the strain Δ*3572*, pNV18-*3572* restored the growth of the knockout strain on DEP and DBP. Compared with the strain Δ*4497*, pNV18-*4497* restored the growth of the knockout strain on DMP, DEP, and DPrP (Fig. S7B and C). Compared with the strain Δ*5299*, pNV18-*5299* restored the growth of the knockout strain (significant difference by *t*-test; Fig. S7D), but growth remained significantly lower than that of the wild type. This incomplete restoration is likely due to the weaker *lac* promoter in pNV18 compared to the native promoter of gene *5299*, which reduced the expression of gene *5299*. These results confirm that protein 5299 impacts the transport of all eight kinds of PAEs.

### Transcription level upregulation of other transporters as each was knocked out

**Fig 4:**
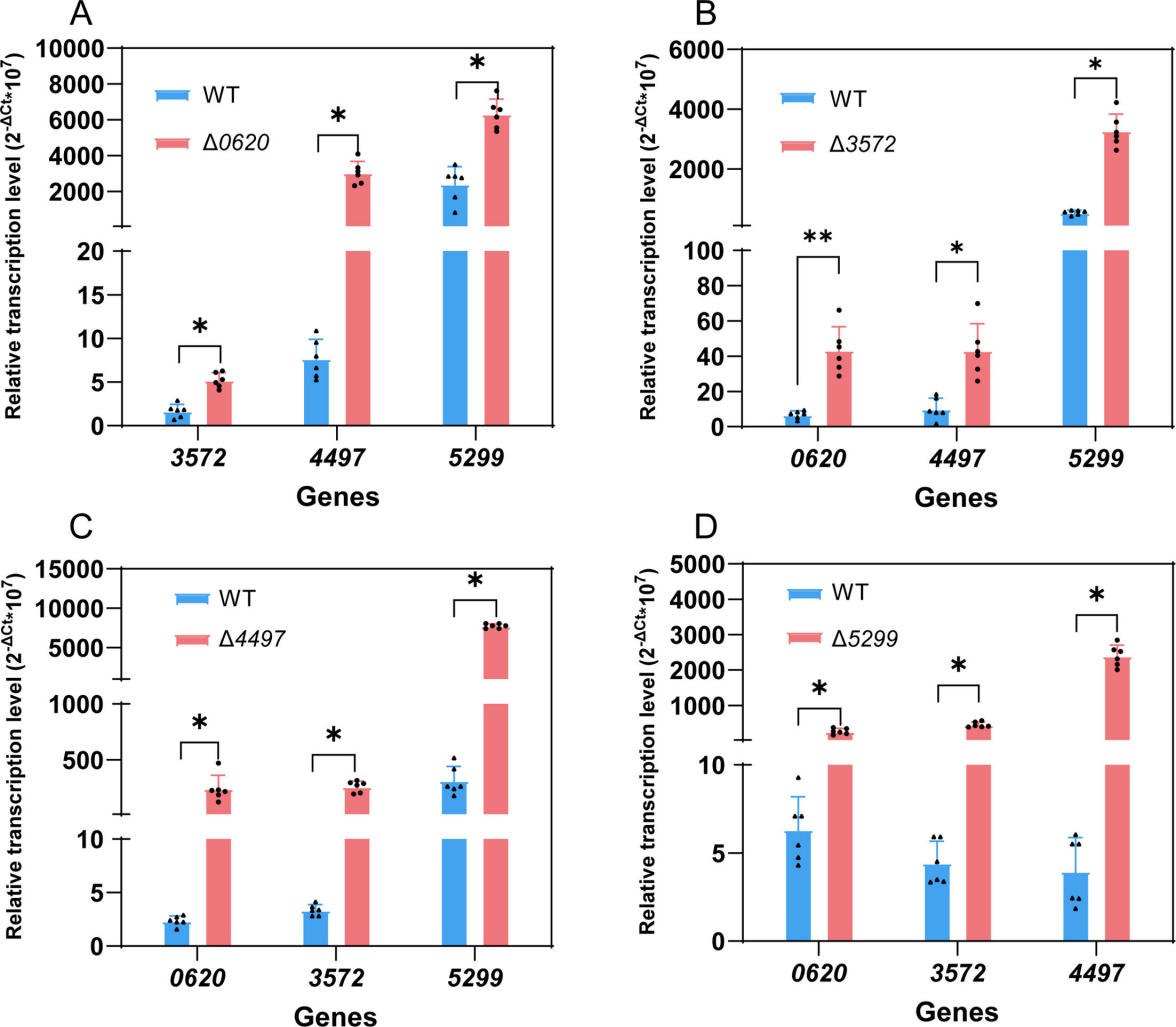
Transcriptional level change (di-n-octyl-phthalate as the sole carbon source, instead of fructose) of transporters 0620, 3572, 4497, and 5299, in mutant strains when any one of them was knocked out. Δ*0620*, *0620* was knocked out (**A**); Δ*3572*, *3572* was knocked out (**B**); strain Δ*4497*, *4497* was knocked out (**C**); and Δ*5299*, *5299* was knocked out (**D**). The data in each column were calculated using the 2^−ΔCT^ threshold cycle (C_T_) method with three technical replicates and two biological replicates. The error bars indicate standard error. Internal reference was 16S rRNA gene. ***, *P* < 0.001; **, 0.001 < *P* < 0.01; *, 0.01 < *P* < 0.05; ns, *P* > 0.05.

The deletion of one membrane protein gene may affect the expression of other transporter genes for physiological function compensation. Thus, changes in the transcription levels of other transporters in the knockout strains were detected. As shown in Fig. 4, each transporter gene knockout resulted in the upregulated transcription of the other three transporter genes, suggesting that the physiological functions of these four membrane proteins may mutually compensate. In detail, the knockout of gene *0620* resulted in the upregulation of the transcriptional levels of *3572*, *4497*, and *5299* upregulation from 1.64 to 5.20, 6.85 to 1760.00, and 2414.00 to 6482.00, respectively (Fig. 4A). The gene *3752* knockout resulted in the transcriptional level of *0620*, *4497*, and *5299* upregulation from 5.76 to 49.61, 10.16 to 48.02, and 530.50 to 3315.00, respectively (Fig. 4B). The knockout of gene *4497* resulted in the upregulation of the transcriptional levels of *0620*, *3572,* and *5299* from 2.35 to 240.70, 3.36 to 259.00, and 312.40 to 7748.00 (Fig. 4C). The knockout of gene *5299* resulted in the upregulation of the transcriptional levels of *0620*, *3572*, and *4497* upregulation from 7.83 to 214.00, 4.44 to 460.50, and 5.69 to 2233.00, respectively (Fig. 4D).

### Evaluation of substrate-binding pocket of PAEs transporters via molecular docking

To explore the mechanism for membrane proteins to transport multiple substrates and the structural characteristics of membrane proteins, the structures and the sizes of active sites of different membrane proteins were evaluated via Swiss-Model and molecular docking.

**Fig 5:**
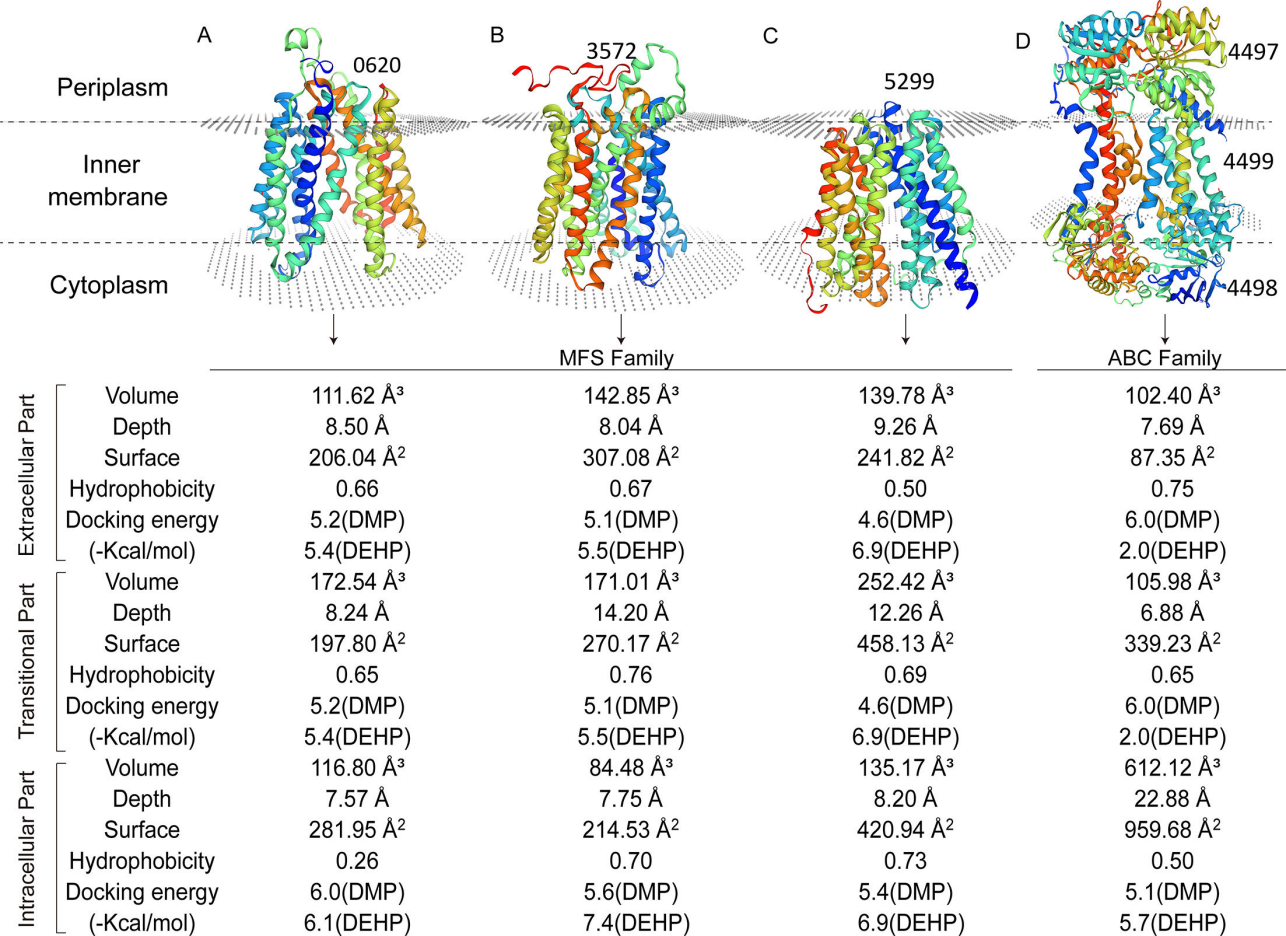
3D Structure, the size of the active center, and docking binding energy of membrane proteins in different locations of the membrane. 3D structure of membrane proteins 0620 (**A**), 3572 (**B**), 5299 (**C**), and 4497-4498-4499 (**D**). 4497, an ABC substrate-binding protein (SBP). 4498, a nucleotide-binding domain (NBD). 4499, a transmembrane domain (TMD). The gray disk represents the cell membrane. The active center and docking binding energy of membrane proteins in the extracellular state, in the transitional state, and in the intracellular state.

Ramachandran plots showed that 99.23%, 98.12%, and 97.88% of amino acid residues belong to the most favored regions for the membrane proteins 0620, 3572, and 5299, respectively (Fig. S8A through C). These results indicated that the backbone conformations of these three membrane protein models are stereochemically reasonable and thus highly reliable. As MFS transporters, each of the proteins 0620, 3572, and 5299 contains 12 transmembrane helices forming a central channel/pocket for substrate transport (Fig. 5A through C). Ramachandran plots also showed that 93.08%, 98.04%, and 95.01% of amino acid residues belong to the most favored regions for the membrane proteins 4497, 4498, and 4499, respectively (Fig. S8D through F). Encoded by a gene cluster, membrane proteins 4497, 4498, and 4499 belong to the ABC transporter family (Fig. 5D). As an ABC substrate-binding protein, 4497 has a concave region for binding small molecules. As a nucleotide-binding domain (NBD), 4498 drives active transport via ATP hydrolysis. As a transmembrane domain (TMD), 4499 forms the substrate channels.

The active center parameters are different for the extramembrane part, transmembrane part, and intramembrane part of each transporter. For example, the active center parameters for the extramembrane part of 5299 are as follows: depth 9.26 Å, surface area 241.82 Å², and volume 139.78 Å³; those for the transmembrane part are as follows: depth 12.26 Å, surface area 458.13 Å², and volume 252.42 Å³; and those for the intramembrane part are as follows: depth 8.52 Å, surface area 420.94 Å², and volume 135.17 Å³ (Fig. 5A). In summary, the active center volume ranking for the extracellular part of the transporters is 3572 > 5299 > 0620 > 4497, while those for the transmembrane part and intramembrane part are 5299 > 0620 > 3572 > 4499, and 4499 > 5299 > 0620 > 3572, respectively. Although the active center volume for the extracellular part of 5299 is not the largest, that of the 5299 transmembrane part (458.13Å³) is much larger than that of 3572 (270.17Å³). The active center volume of the membrane protein 5299 intramembrane part is also larger than that of membrane proteins 0620 and 3572. Although the size of the intracellular active center of 4499 is much larger than those of 0620, 3572, and 5299, 4499 is a channel protein belonging to the ABC family, and the transport capacity of 4499 is affected by the extracellular 4497 protein (Fig. 5D).

Based on their larger active centers and higher transport efficiencies compared to proteins 0620 and 3572 (Fig. 3 and 5), membrane protein 5299 and the ABC complex 4497-4498-4499 were selected as representatives of the MFS and ABC families for investigating PAEs transport mechanisms, respectively.

**Fig 6:**
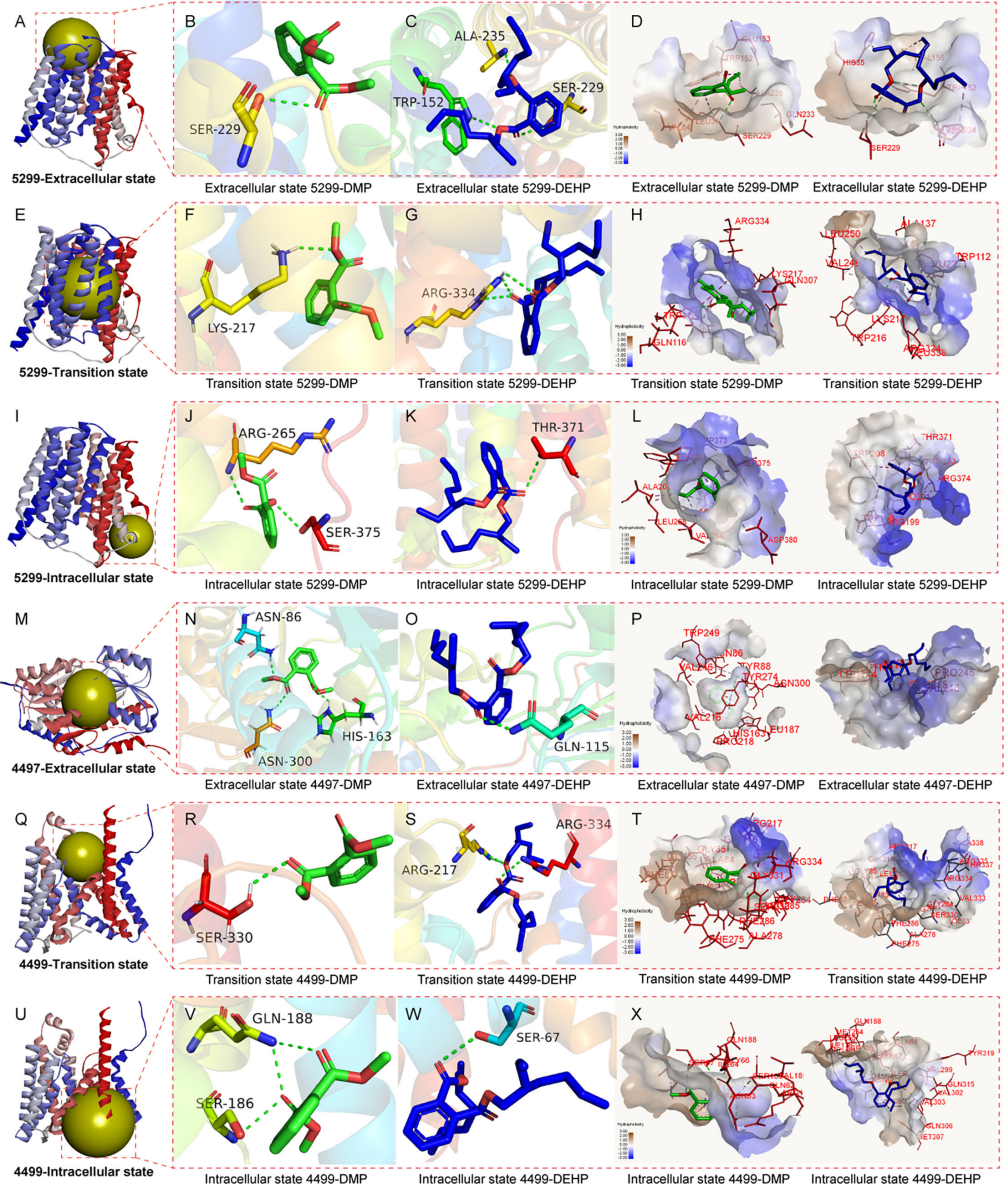
Substrate binding analysis of membrane proteins 4497, 4499, and 5299 in extracellular state, transitional state, and intracellular state. The active center position of membrane protein 5299 in the extracellular state (**A**), in the transition state (**E**), and in the intracellular state (**I**). Membrane protein 5299 and DMP (**B**), DEHP (**C**), molecular docking in the initial state, DMP (**F**), DEHP (**G**) molecular docking in the transition state, DMP (**J**), and DEHP (**K**) molecular docking in the intracellular state. The hydrophobic receptor surface of membrane protein 5299 and DMP, DEHP in the extracellular state (**D**), in the transition state (**H**), and in the intracellular state (**L**). The active center position of membrane protein 4497 in the extracellular state (**M**), 4499 in the transition state (**Q**), and 4499 in the intracellular state (**U**). Membrane protein 4497 and DMP (**N**), DEHP (**O**), molecular docking in the extracellular state, DMP (**R**), DEHP (**S**), molecular docking in the transition state, DMP (**V**), and DEHP (**W**) molecular docking in the intracellular state. The hydrophobic receptor surface of membrane protein 5299 and DMP, DEHP in the extracellular state (**P**), in the transition state (**T**), and in the intracellular state (**X**).

Protein 5299 exhibited a more significant effect on DMP and DEHP degradation than on other PAEs; thus, molecular docking of 5299 with DMP and DEHP was performed. PAEs are predicted to bind to the outer side of the MFS transporter (Fig. 6A), enter its central cavity (Fig. 6E), and then enter the cell (Fig. 6I). In the extracellular part, DMP binds via a hydrogen bond with Ser229 (Fig. 6B). In the transition state, Lys217 forms a hydrogen bond to facilitate transport (Fig. 6F). In the intracellular part, both Ser375 and Arg265 form hydrogen bonds with DMP, stabilizing the small molecule within the pocket (Fig. 6J). For DEHP, 5299 forms a hydrogen bond network with Ala235 and Trp152, maintaining the stability of the substrate-protein complex in the extracellular state (Fig. 6C); in the channel, it forms a hydrogen bond with Arg334, enabling cellular entry (Fig. 6G). DEHP is stabilized within the pocket by Thr374 in the intracellular part (Fig. 6K). The molecular docking results showed that substrates with different hydrophobicity exhibited distinct hydrophobic surface profiles at the same position (Fig. 6D,H and L ). The hydrophobicity of DEHP was higher than that of DMP, so the number of hydrophobic amino acids around DEHP was greater than that around DMP. Blue indicates hydrophilicity, so the blue color around DEHP is weaker than that around DMP.

Based on substrate degradation assays, the effect of membrane protein 4497 on the degradation of DMP was much greater than that of DEHP (Fig. 3). As a channel protein, 4499 forms a complex with 4497 outside the cell to co-transport PAEs, and they have different active centers during different transport processes (Fig. 6M, Q and U ). 4498 is a protein that provides ATP and does not directly participate in PAEs transport (Fig. 5D). Molecular docking with DMP and DEHP was conducted to elucidate their differential degradation. In the extracellular state, DMP formed hydrogen bonds with residues His163, Asn300, and Asn85 of 4497, stabilizing its binding deep within the active pocket (Fig. 6N). In contrast, DEHP formed only one hydrogen bond, via Gln115, and bound near the entrance of the pocket (Fig. 6O). Hydrophobic surface analysis further confirmed that DMP penetrated the active center, while DEHP remained at the entrance (Fig. 6P). In both the transition and intracellular parts, Ser and Arg residues formed hydrogen bonds with PAEs (Fig. 6R, S, V and W), indicating their conserved role in MFS and ABC transporter-mediated PAE transport. Hydrophobic surface profiles of DMP and DEHP showed no significant differences in these states (Fig. 6T and X), suggesting that the extracellular substrate-binding protein primarily determines substrate specificity in ABC transport.

### Enhanced PAE degradation by transporter overexpression strains

**Fig 7:**
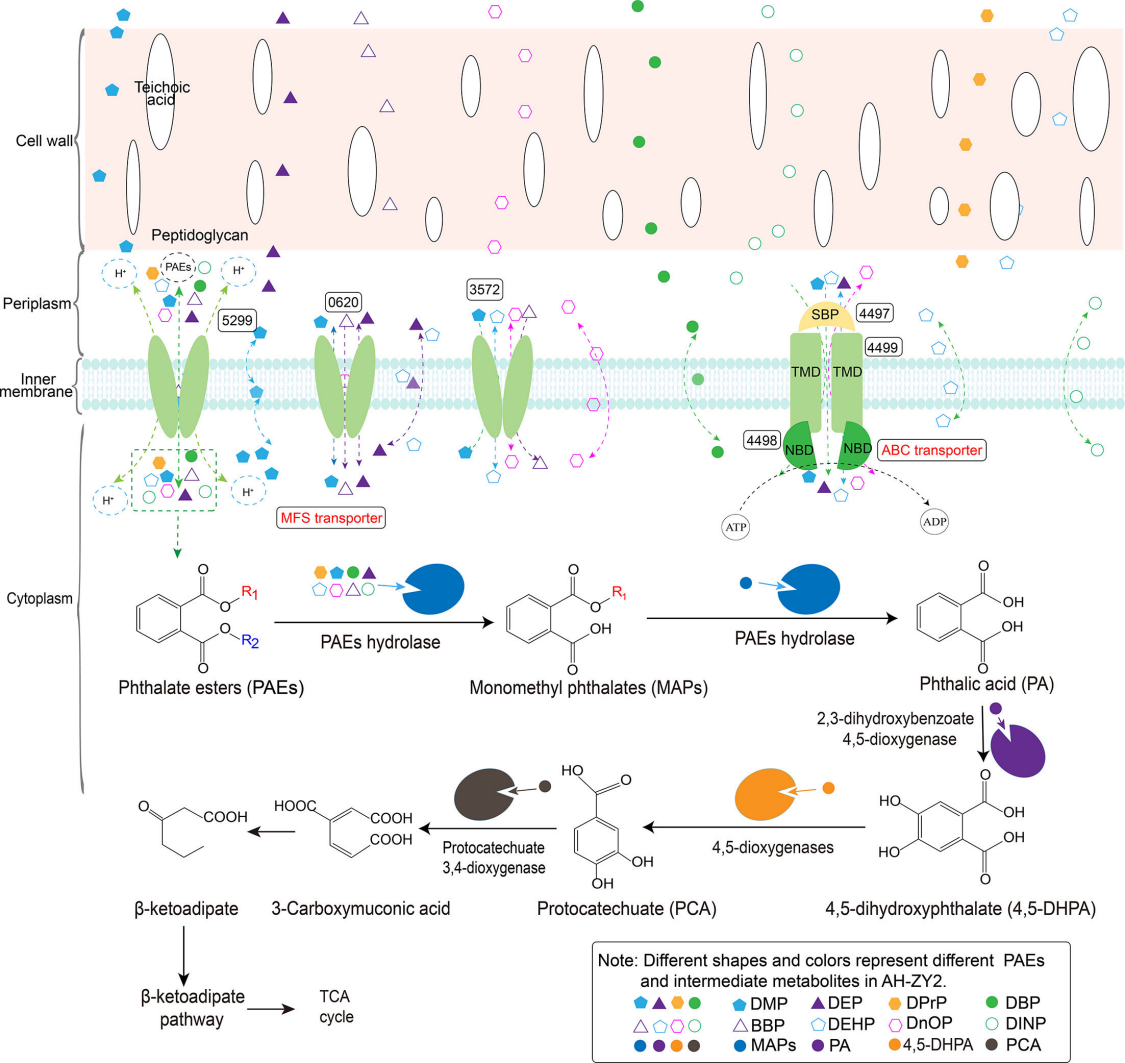
Proposed pathways of multiple PAEs transport and metabolism in *Rhodococcus* sp. AH-ZY2.

**Fig 8:**
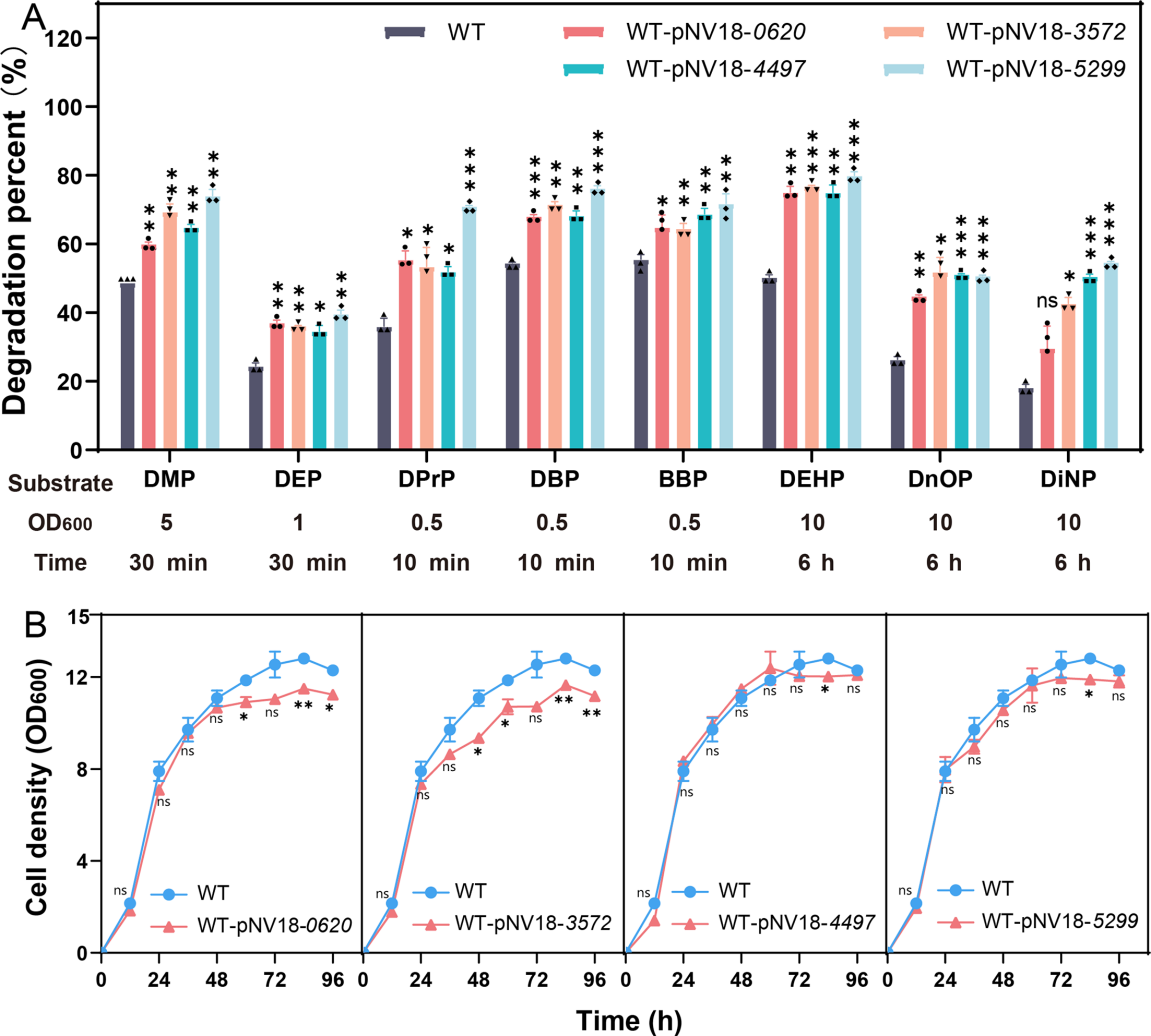
Enhanced degradation of eight individual PAEs (800 mg/L) in PBS by the whole cells of membrane transporters overexpression strains (**A**) and effect on their growth in LB (**B**). Bars represent mean ± SD from three independent experiments (*n* = 3). ***, *P* < 0.001; **, 0.001 < *P* < 0.01; *, 0.01 < *P* < 0.05; ns, *P* > 0.05.

PAE’s entry into AH-ZY2 and its metabolic pathway were predicted as shown in Fig. 7. When PAEs enter the cell, PAEs need to first traverse the cell wall to enter the periplasmic space, then cross the cell membrane into the intracellular compartment, where they are captured and degraded by endogenous esterases. To enhance PAEs degradation, the genes of membrane proteins 0620, 3572, 5299, and 4497 were transformed into strain AH-ZY2 via the overexpression plasmid pNV18 (WT as control). The whole cells of the resultant recombinant strains were used to evaluate their PAEs degradation ability. For the single PAEs, the overexpression of membrane protein genes 0620, 3572, 5299, and 4497 all enhanced the degradation of different PAEs. Among them, the overexpression of 5299 significantly increased the degradation efficiency of all tested PAEs, DMP (from 48.76% to 74.08%), DEP (from 24.49% to 39.66%), DPrP (from 36.05% to 71.04%), DBP (from 54.58% to 76.28%), BBP (from 55.60% to 71.83%), DEHP (from 50.30% to 79.99%), DnOP (from 26.38% to 50.81%), and DiNP (from 18.21% to 54.73%) (Fig. 8A). The WT-pNV18-*5299* strain showed higher degradation efficiency for DMP, DEP, DPrP, DBP, BBP, DEHP, DnOP, and DiNP than all the other membrane protein-overexpressing strains (Fig. 8A). The transformation of pNV18-*0620* and pNV18-*3572* plasmids resulted in impaired growth of the wild-type strain in LB medium (Fig. 8B). In contrast, no significant impact of pNV18-*4497* and pNV18-*5299* plasmid transformation on the growth profile of WT cultured in LB was detected (Fig. 8B).

**Fig 9:**
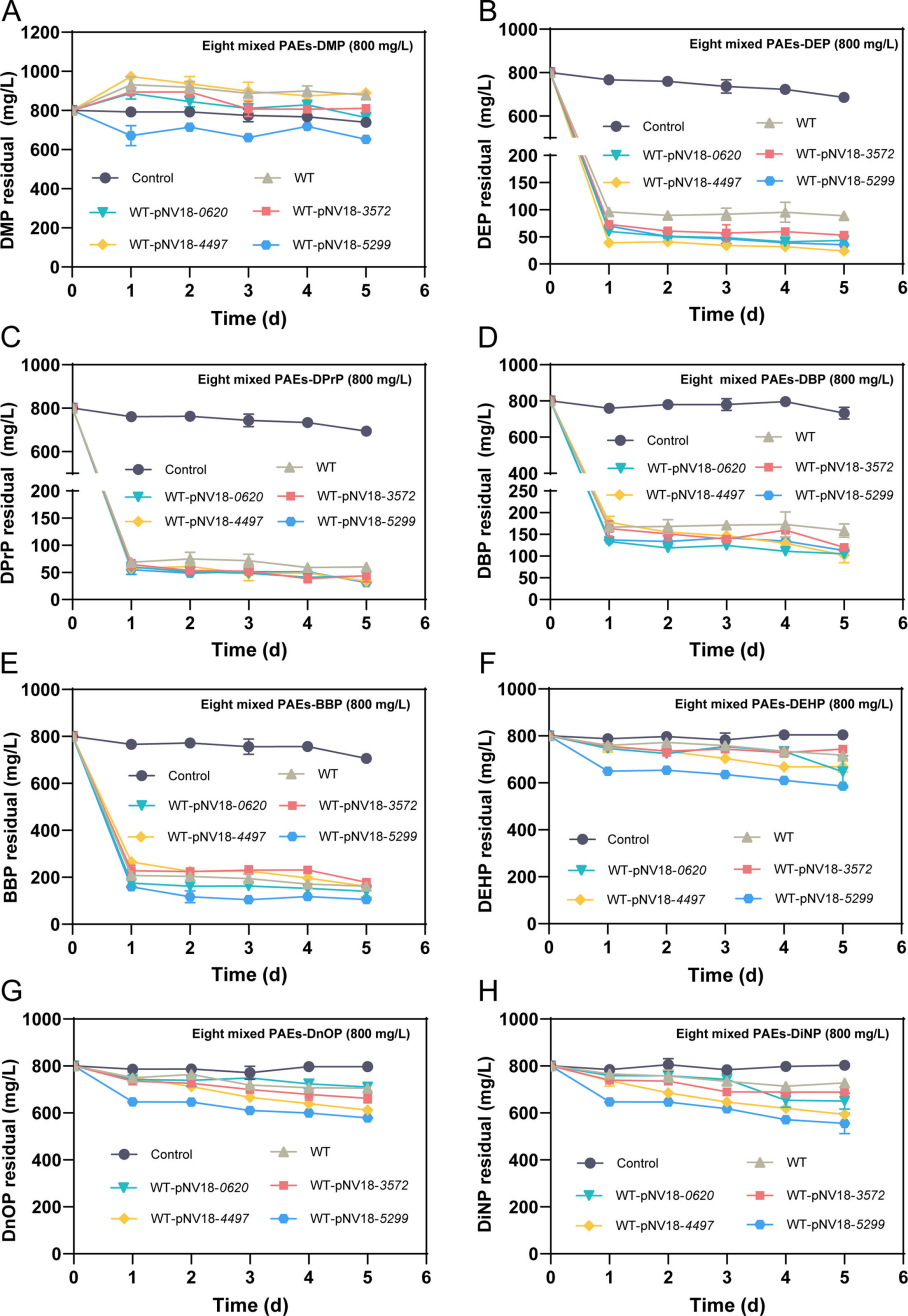
Degradation curves of eight mixed PAEs (each at 800 mg/L) by whole cells of WT and WT-pNV18-*0620*, pNV18-*3572*, pNV18-*4497*, and pNV18-*5299*. DMP (**A**), DEP (**B**), DPrP (**C**), DBP (**D**), BBP (**E**), DEHP (**F**), DnOP (**G**), and DiNP (**H**). Bars represent mean ± SD from three independent experiments (*n* = 3).

Compared to the WT, the engineered strain WT-pNV18-*5299* demonstrated enhanced degradation of eight kinds of mixed PAEs. During the degradation of mixed PAEs, the WT strain exhibited significant accumulation of the intermediate product DMP (Fig. 9A). In comparison with the engineered strains WT-pNV18-*0620*, WT-pNV18-*3572,* and WT-pNV18-*4497* for mixed PAEs degradation, WT-pNV18-*5299* significantly reduced DMP accumulation and markedly improved the degradation efficiency of BBP, DEHP, DnOP, and DiNP (Fig. 9). In the degradation process of mixed PAEs, DMP was degraded, but the generation of new DMP from other PAEs offset this degradation, resulting in no significant change in the DMP concentration in the system (Fig. 9A).

## DISCUSSION

The transcriptional levels of multiple MFS and ABC transport protein genes in AH-ZY2 were significantly upregulated in response to PAEs, which were identified via genomics and transcriptomics analyses. Literature results show that aromatic compound-transporting MFS transporters belong to the MHS, AAHS, and DHA1 families (17, 28, 34), with no reports of the NNP or UMF families being involved (Table S1). Membrane proteins 5299, 4497, and 0620 exhibit low sequence similarity to all known functional protein families (Fig. 1). It is speculated that membrane proteins 0620, 4497, and 5299 belong to new membrane protein families that have not been reported to transport PAEs based on phylogenetic tree, TCDB, and NCBI database analyses.

There is only one report on the transporters for PAEs degradation. Hara et al. reported a novel ABC transporter PatB, which is a channel protein. The *patB* knockout strain of *Rhodococcus jostii* RHA1 lost its ability to grow on phthalate, monomethyl phthalate, monobutyl phthalate, and monohexyl phthalate (10). However, *Rhodococcus jostii* RHA1 could not grow on most dialkyl PAEs. In our study, *Rhodococcus* sp. AH-ZY2 exhibited the ability to degrade most dialkyl PAEs. Thus, their membrane transporters should be different in diversity and characteristics. In AH-ZY2, the ABC transporter 4497 is a substrate-binding protein, the knockout of which reduced the degradation efficiency of DMP, DEP, BBP, DEHP, DnOP, and DiNP. No reported ABC transporters for transporting aromatic compounds were found to be similar to 4497 via NCBI comparison. As for MFS transporters, they have been widely reported to be utilized for xenobiotic degradation (Table S1). In AH-ZY2, MFS transporter 0620 belongs to the NNP family, none of which has been reported for pollutant transport. MFS transporter 3572 belongs to the MHS family and has 33.44% similarity to another MHS family transporter PcaT, which is involved in the ferulic acid degradation (34). MFS transporter 5299 provides the first functional insight into PAEs transportation into the uncharacterized UMF22 family. The above-mentioned findings revealed a novel function of environmental pollutant transport within the MFS superfamily.

The key roles of these proteins in bacterial PAEs cell growth and degradation efficiency were revealed by knocking out multiple membrane protein genes (Δ*0620*, Δ*3572*, Δ*5299*, and Δ*4497*). The PAEs degradation experimental results of the knockout strains showed that different membrane proteins exhibited specific responses to different types of PAEs, suggesting that these proteins may be involved in the recognition, uptake, and transport of substrates. For instance, the Δ*5299* mutant showed significant growth inhibition and decreased degradation efficiency across all tested PAEs, implying that this protein may play a core role in PAEs transport and primary metabolism (Fig. 2 and 3).

Knockout of specific membrane protein genes also affected the transcriptional levels of other transporter genes, indicating the existence of a regulatory network to cope with the loss of membrane protein function. For instance, the upregulated expression of other membrane protein genes in the Δ*5299* mutant may represent a compensatory mechanism to maintain substrate transport capability. Such coordinated regulation is common when bacteria respond to environmental stress (11).

The analysis of the active centers of different membrane proteins showed that the larger the active center of the membrane protein, the higher the hydrophobicity of the substrate, and the more favorable the transport of the substrate. The FadL protein has a longer extracellular loop, a larger functional center, and can bind more hydrophobic and larger substrates (16). It is speculated that the size of the active center of a membrane protein is related to its substrate diversity (15).

The lower the binding energy in molecular docking, the more likely the reaction is to occur, meaning that the site is more prone to binding with small molecules. In the extracellular parts, the binding energies of membrane proteins 0620, 3572, and 5299 to DMP and DEHP are higher than those in the intracellular parts, suggesting that DMP and DEHP are more easily captured extracellularly and tend to be transported into the cell (Fig. 5). Proteins 4497, 4498, and 4499 are components of an ABC transporter complex. In this system, protein 4497 acts as both the substrate-binding protein and the transmembrane channel, while protein 4498 provides energy via ATP hydrolysis. In contrast, MFS transporters combine substrate binding and channel formation into a single polypeptide and utilize the proton motive force for transport. Both ABC and MFS transporters are vital for cellular processes such as metabolism, detoxification, and pollutant degradation (28, 43, 44). DEHP has higher hydrophobicity than DMP. The magnitude of docking binding energies at different locations indicates that, whether in the extracellular, transition, or intracellular state, the binding energies of proteins 0620, 3572, 5299, and 4497 for DEHP are consistently lower than those for DMP (Fig. 5). This indicates that the hydrophobicity of the substrate influences the transport ability of membrane proteins (16).

The Δ*5299* mutant showed a significant reduction in the degradation efficiency of all tested PAEs. The mechanistic role of this transporter, especially in PAEs recognition and initial binding, needs to be revealed. The large substrate-binding pocket (especially the extracellular part volume: 252.42 Å³) of transporter 5299 provides the structural foundation for its recognition of diverse PAEs (Fig. 5). The results of molecular docking show that the process of PAEs transport by MFS transporters was consistent with the “rocker switch” model. Substrates first bind to the extracellular side of the membrane protein, stabilizing within its active pocket (45), opening the extracellular side while closing the intracellular side. Molecular docking further revealed the molecular mechanism of recognition in the extracellular part, key residues, specifically Ser229 for DMP and a hydrogen-bonding network involving Ala235 and Trp152 for DEHP, directly mediate the initial binding and “capture” of distinct PAEs substrates (Fig. 6). This favorable initial binding is thermodynamically supported by the calculated low binding energies. Furthermore, in contrast to ABC transporters like 4497-4498-4499, which rely on discrete, specialized substrate-binding proteins, 5299 integrates the functions of substrate recognition, binding, and translocation within a single polypeptide. Notably, Ser and Arg are crucial for substrate transport across various protein states, forming hydrogen bonds during the process. For instance, in NarU, Ser residues form a polar layer essential for its outward-closed conformation (46). Mutating Arg to Ala in GLUT4 reduces glucose transport by 10% (47). Structural studies of NarU and NarK highlight Arg’s key role in coordinating nitrate and mediating the antiport mechanism (48, 49). Thus, Ser and Arg are fundamental to the transport of diverse substrates, including PAEs. Future work could employ site-directed mutagenesis and isotope tracing technology to confirm the significance of these residues and to enhance membrane protein transport efficiency and to improve PAEs degradation.

Based on the degradation phenotypes, membrane proteins may be one of the rate-limiting steps for PAEs degradation in strain AH-ZY2 (42, 50, 51). Therefore, in this study, multiple engineered strains were constructed, and their degradation performance under single and mixed PAEs conditions was evaluated. The results showed that the engineered strain WT-pNV18-*5299* could significantly enhance the degradation efficiency of multiple PAEs, particularly by reducing the accumulation of the intermediate product DMP in mixed systems, indicating that this protein plays a key role in the transmembrane transport of PAEs. Furthermore, under mixed PAEs conditions, the engineered strain maintained high degradation activity and reduced intermediate product accumulation, suggesting that 5299 may possess broad substrate specificity or can coordinate the balance of transport when multiple substrates coexist. Notably, the overexpression of 0620 and 3572 led to growth impairment, which could be linked to an imbalance in energy allocation or effects on membrane integrity. This suggests that subsequent engineering efforts need to comprehensively consider the overall impact of protein gene expression on cellular physiology. Research has also noted that the excessive expression of exogenous membrane proteins may trigger membrane stress, affecting strain stability and adaptability (52).

In summary, this study identified several novel membrane proteins involved in the transport of PAEs. Substrate degradation assays validated the substrate specificity of these transporters, and molecular docking provided insights into the mechanisms enabling multiple substrate transport. Furthermore, comparative analyses of the enhanced expression of different membrane protein genes revealed that enhancing membrane protein 5299 most effectively improved PAEs degradation efficiency. This study paves the way for constructing a membrane protein engineering strain to enhance PAEs degradation and identifies key protein targets for elucidating the transport mechanisms of other xenobiotics.

However, overexpression of some membrane transporter genes also exhibited a slight inhibition on the growth of the engineering strains in LB, suggesting that some membrane transport proteins may not only transport PAEs but also possess certain uncharacterized functions. Furthermore, the competitive fitness and ecological safety of the engineered strain in natural environments are uncertain. Finally, substrate concentrations in natural settings may be suboptimal, potentially failing to induce or even inhibiting transporter function. All the above-mentioned issues are challenges for the application of the membrane transport protein engineering strains in real-world bioremediation scenarios.

## MATERIALS AND METHODS

### Chemicals, media, and strains

DMP (dimethyl phthalate), DEP (diethyl phthalate), DPrP (dipropyl phthalate), DBP (dibutyl phthalate), BBP (benzyl butyl phthalate), DEHP (Bis(2-ethylhexyl) phthalate), DnOP (di-n-octyl-phthalate), DiNP (diisononyl phthalate), PA (phthalic acid), BA (benzoic acid), PCA (protocatechuic acid), and MBP (monobutyl butyl phthalate) with 99% purity were purchased from Aladdin Biochemical Technology Co., Ltd. (Shanghai, China). The basic inorganic salt medium (BSM) contains K_2_HPO_4_·3H_2_O 1.0 g/L, NaCl 1.0 g/L, (NH_4_)_2_SO_4_ 0.5 g/L, MgSO_4_·7H_2_O 0.4 g/L, CaCl_2_ 0.0755 g/L, and FeCl_3_ 0.0143 g/L. The LB medium: NaCl 10.0 g/L, peptone 10.0 g/L, yeast powder 5.0 g/L. For kanamycin, the concentration of the stock solution is 50 mg/mL, and the working concentration is 250 μg/mL. *E. coli* WM3064, *E. coli* DH5α, and the pK18*mobsacB* plasmid are from our laboratory. The pNV18 plasmid is from Shanghai NovoPro Biotechnology Co., Ltd.

### Mining of membrane protein genes for PAEs transport in AH-ZY2

Fructose (control group), DBP (experimental group), DnOP (experimental group 1), and mixed PAEs of DMP, DEP, DPrP, DBP, BBP, DEHP, DnOP, and DiNP (experimental group 2) were used as the sole carbon sources, respectively, for strains transcriptome analysis. When the broth OD_600_ reached 0.6–0.8, cells were harvested via centrifugation (8,000 rpm), then stored at −80°C, and shipped on dry ice for transcriptome sequencing (Hangzhou Mingke). The expression levels of MFS and ABC transporter genes in AH-ZY2 under different PAEs were detected, and the highly expressed membrane protein genes across all experimental groups were selected for knockout verification.

The families of membrane proteins from AH-ZY2 were predicted using the TCDB database (https://www.tcdb.org/progs/blast.php). Amino acid sequences of MFS and ABC transporters were obtained through a literature review (37) and TCDB. The similarities of the proteins obtained from TCDB were compared via NCBI. A phylogenetic tree was constructed using MEGA X software (https://www.megasoftware.net/dload_win_gui) and refined with Adobe Illustrator 2023.

### RNA sequencing and library preparation

RNA library construction and sequencing were performed using the Illumina TruSeq Stranded RNA Sample Prep Kit, starting with 5 μg of total RNA. The procedure included ribosomal RNA depletion with Ribo-Zero, mRNA fragmentation, synthesis of strand-specific cDNA (with dUTP incorporation for second-strand marking), adapter ligation, size selection of 200–300 bp fragments via gel electrophoresis, and PCR amplification for 15 cycles. After quantification and pooling, paired-end sequencing (2 × 150 bp) was carried out on an Illumina HiSeq platform. The overall quality of the RNA-seq data was assessed using RSeQC v2.6.1 (https://rseqc.sourceforge.net/), which performed sequencing saturation analysis and gene coverage uniformity analysis.

### Construction of AH-ZY2 membrane protein gene knockout strains

SnapGene 6.0.2 software (https://www.snapgene.com/) was used to design primers (Table S5). The pK18*mobsacB* suicide plasmid was linearized via reverse PCR using primers containing *Bam*HI and *Hin*dIII restriction sites. Upstream and downstream homologous arms (~500 bp each) of the target gene were amplified with designed primers. Target homologous arms and linearized plasmid were then fused via fusion PCR and one-step cloning (Nanjing Vazyme, China) to construct the knockout vector, which was transformed into DH5α. The vector was extracted for heat-shock transformation into WM3064. Finally, the knockout vector in WM3064 was transferred into wild-type AH-ZY2 by conjugation. Primary screening was verified by colony PCR, followed by secondary screening on 15% sucrose plates. Successful knockout stains were confirmed and selected via colony PCR.

### Growth, degradation, and extracellular and intracellular concentration measurement

The AH-ZY2 wild strain and its mutant strains were, respectively, transferred into 100 mL of LB medium and cultured at 37°C, 220 rpm until the OD_600_ reached 0.8–1.0 (logarithmic phase). The cells were harvested by centrifugation at 8,000 rpm for 5 min and washed with sterile water three times. Then, the cell pellets were resuspended in 100 mL sterile water and inoculated (2%, vol/vol) into 50 mL of BSM containing 500 mg/L of PAEs (DMP, DEP, DPrP, DBP, BBP, DEHP, DnOP, and DiNP, respectively) as the sole carbon source. After culturing at 37°C and 220 rpm for 48 h, the residual PAEs and the growth curves were measured, respectively.

The solubility of PAEs in water is low and varies with the length of their side chains. The insoluble PAEs in the aqueous growth medium suspend at the water-air interface. Thus, all the residual PAEs required full extraction before detection using high-performance liquid chromatography (HPLC). An equal volume of dichloromethane was added to the whole-flask BSM broth with cells and to the control BSM broth without cells as a reference to ensure the accuracy of PAE quantification. The mixture was vortexed for 10 min and transferred to a separatory funnel. After standing for 10 min, the lower organic phase containing PAEs was collected, which was then evaporated using a rotary evaporator, and the residue was redissolved in 5 mL of methanol. The solution was thoroughly mixed, filtered through a 0.20 μm organic membrane before analysis via HPLC.

For the growth curves in LB, the OD_600_ of the wild-type bacteria and knockout bacteria was measured every 12 h using an ultraviolet spectrophotometer (UV-5200, Shanghai Yuanxi Instrument Co., China). All samples were in triplicate, and the growth curves were plotted using GraphPad Prism 9 (https://www.graphpad.com/scientific-software/prism/).

For the whole-cell degradation test, a higher concentration of PAEs (800 mg/L) than for growing-cell degradation in BSM (500 mg/L) was set, because a rapid PAEs degradation rate by whole cell than by growing cell may result in no PAEs being detected after 4-h degradation. Meanwhile, the concentration of PAEs in the environment may be at the level same to our set concentration (53). For example, high concentrations of DBP (599 ± 34 mg/kg) and DEHP (1458 ± 142 mg/kg) have been detected in wastewater-contaminated soil in São Paulo, Brazil (54). Overall, 1532.987 mg/kg PAEs were detected in cotton field soils in Xinjiang, China (55). WT and knockout strains were cultured in LB till the OD_600_ of the broth reached 0.8–1.0 (logarithmic phase) and then harvested by centrifugation at 8,000 rpm for 5 min. The pellet was washed twice with PBS and resuspended until the OD_600_ reached 10 for the following resting cells experiments. Meanwhile, a 10 g/L PAE (DMP/DEP/DPrP/DBP/BBP/DEHP/DnOP/DiNP) stock solution was prepared in methanol. In experimental groups, 920 μL of bacterial suspension was mixed with 80 μL of PAEs stock solution (10 g/L) in a 1 mL system (final concentration 800 mg/L PAEs). In the control groups, the bacterial suspension was replaced by PBS. After 4-h incubation at 37°C and 220 rpm, cells were harvested via centrifugation, and then extracellular PAEs were extracted via mixing supernatants (1 mL) with methanol (3 mL) and vortexing for 1 h. After the cells were broken via ultrasonication and then centrifugation, intracellular PAEs were extracted via a mixture of supernatants (1 mL) with 3 mL of methanol and vortexed for 1 h. Meanwhile, the attached PAEs were extracted via mixture precipitate with 4 mL of methanol. All samples were analyzed in triplicate.

### Plasmid complementation and phenotype

Since the pNV18 plasmid is a *Rhodococcus* sp. specific plasmid and can express protein genes in *Rhodococcus* sp., Membrane transporter encoding genes *0620, 3572, 5299*, and *4497* were cloned into the complementation plasmid pNV18, and the resultant recombinant plasmids were transformed into the knockout strains Δ*0620,* Δ*3572,* Δ*5299*, and Δ*4497,* respectively. Then the WT and complemented strains were cultured in LB containing 50 mg/L streptomycin until the broth OD_600_ reached 0.8–1.0. The cells were harvested by centrifugation, then washed and resuspended with an equal volume of PBS. The resuspension of cells was inoculated (2%, vol/vol) into BSM containing 500 mg/L of PAEs (DMP, DEP, DPrP, DBP, BBP, DEHP, DnOP, and DiNP, respectively) as the sole carbon source. After culturing for 48 h, the broth OD_600_ was measured using a spectrophotometer (UV-5200, Shanghai Yuanxi Instrument Co., China).

### Detection of transcription levels of gene *0620*, *3572*, *4497*, and *5299*

WT and mutant strains Δ*0620,* Δ*3572,* Δ*5299*, and Δ*4497* were cultured, respectively, in 50 mL of basal salts medium containing 500 mg/L of DnOP as the sole carbon source. When the broth OD_600_ reached 0.6 (logarithmic phase), cells were harvested by centrifugation (8,000 rpm, 5 min), followed by total RNA extraction. After DNase treatment (HiScript II qRT SuperMix, Vazyme), cDNA was synthesized. Transporter gene transcription levels were quantified via RT-qPCR (CFX Connect System, Bio-Rad) using the 16S rRNA as the reference gene. The relative expression level was calculated using the 2⁻^ΔCt^ method with three biological and technical replicates.

### Homology modeling and molecular docking

Membrane protein 5299 in the MFS family and membrane protein 4497 in the ABC family both could transport multiple PAEs. Therefore, molecular docking of membrane proteins 5299 and 4497 with PAEs was performed to determine whether there were differences in the transport mechanisms between the MFS and ABC families. The 3D structures of 0620, 3572, 5299, and the 4497-4498-4499 complex were predicted via Swiss-Model (https://swissmodel.expasy.org/interactive), with template PDB files downloaded from the PDB database (https://www.rcsb.org/) and visualized using Pymol and Discovery Studio. Based on literature for the possible active center location of the transporter and combining the active pocket predicted by DoGSiteScorer (https://proteins.plus/), the active centers of transporters 5299 and 4497 were speculated. To assess binding energies and interaction patterns between membrane proteins and DMP/DEHP, Autodock Vina 1.2.2 was used for protein-ligand docking (56). 3D structures of PAEs were generated via ChemDraw 20.0.0.41 (https://www.chemdraw.com.cn/), optimized via molecular dynamics, and defined as ligands. Protein and ligand files were prepared by converting to PDBQT format, removing water molecules, adding polar hydrogens, and centering a 25 Å × 25 Å × 25 Å grid box over the protein domains to allow free molecular movement. Docking results were visualized via Discovery Studio (https://www.3ds.com/products/biovia/discovery-studio) and Pymol (https://pymol.org/).

### Enhanced PAE degradation by transporter overexpression strains

Membrane proteins 0620, 3572, 5299, and 4497, which are involved in the transport of all eight kinds of tested PAEs, were identified via knockout and complementation experiments. To enhance the PAEs degradation capability of the WT, membrane protein genes *0620*, *3572*, *5299*, and *4497* were overexpressed in WT strains via overexpression plasmids.

Using the genomic DNA of the WT strain as a template, gene fragments encoding *0620*, *3572*, *5299*, and *4497* were amplified with designed primers (Table S5), and then cloned into plasmid pNV18 via a one-step cloning kit. The resultant recombinant plasmids were introduced into the WT strain via electroporation. Successful plasmid transformation was confirmed by colony PCR using specific primers.

Five strains, WT-pNV18-*0620,* WT-pNV18-*3572*, WT-pNV18-*5299*, WT-pNV18-*4497*, and the WT, were cultured in 200 mL of LB containing 50 mg/L of streptomycin until the OD_600_ of the broth reached 1.6. The cells were harvested by centrifugation, then washed and resuspended with 1× PBS, affording a concentration of whole cells as OD_600_ was set to 0.5, 1, 5, or 10, for the degradation of single or mixed PAEs, respectively. Since the WT exhibits varying degradation efficiencies for different PAEs, different OD_600_ values of the whole-cell suspension and different reaction times were designed for different PAEs (for single PAE degradation, DMP [OD_600_ 5, reaction 30 min], DEP [OD_600_ 1, reaction 30 min], DPrP, DBP, and BBP [OD_600_ 0.5, reaction 10 min], DEHP, DnOP, and DiNP [OD_600_ 10, reaction 6 h]; for mixed PAE degradation, DMP, DEP, DPrP, DBP, BBP, DEHP, DnOP, and DiNP [OD_600_ 10, reaction 5 d]). In a 1 mL reaction system, 920 μL of bacterial suspension was mixed with 80 μL of PAEs stock (10 g/L for single PAEs or 80 g/L for mixed PAEs in methanol) in a 10 mL Eppendorf tube as the experimental group, while PBS replaced the bacterial suspension as the control group. After degradation of single and mixed PAEs at 37°C and 220 rpm for a specific duration, all samples were collected at once for single PAEs or once a day for mixed PAEs, then extracted with three volumes of methanol, vortexed for 10 min, and finally centrifuged. The supernatant was filtered through a 0.20 μm organic membrane before HPLC analysis.

### High performance liquid phase (HPLC) detection method of PAEs

In this study, a standard curve method was used to calculate the concentration of PAEs (Fig. S9). An Eclipse XDB-C18 column (150 mm × 4.6 mm, 5 μm) was utilized with the UV detection wavelength set at 235 nm. For single PAEs detection, 100% methanol was used as the mobile phase. For mixed PAEs detection, mobile phases A (water) and B (methanol) were employed with the following gradient elution program: 0.00–7.00 min, 75% B; 7.00–9.00 min, 75%–100% B; and 9.00–16.00 min, 100% B. The flow rate was 1 mL/min, the injection volume was 10 μL, and the column temperature was 30°C.

## Data Availability

All RNA-seq data were submitted to the SRA under BioProject number PRJNA1053484. Additional NCBI accession numbers are as follows: for gene AH-ZY2-0620, WML65804.1; for AH-ZY2-3572, WML65818.1; for AH-ZY2-4497, WML64558.1; and for AH-ZY2-5299, WML66394.1. Data analysis was performed using GraphPad Prism 9 (https://www.graphpad.com/scientific-software/prism/). The significance tests used in this study included negative binomial regression analysis for transcriptomic data and one-way ANOVA for other data.

## SUPPLEMENTAL MATERIAL

Supplemental material aem.02526-25-s0001.docx Fig. S1 to S9; Tables S1 to S5.

ASM does not own the copyrights to Supplemental Material that may be linked to, or accessed through, an article. The authors have granted ASM a non-exclusive, world-wide license to publish the Supplemental Material files. Please contact the corresponding author directly for reuse.

## References

[1] Xu YL, Sun YQ, Lei M, Hou J. 2024. Phthalates contamination in sediments: a review of sources, influencing factors, benthic toxicity, and removal strategies. Environ Pollut 344:123389. doi:10.1016/j.envpol.2024.123389 38246215

[2] Wang L, Liu YY, Zhang YW, Chen SW, Zhang NN, Wang ZF, Liu HF. 2023. Estimation and potential ecological risk assessment of multiphase PAEs in mangrove wetlands in Dongzhai Harbor, Hainan. Sci Total Environ 870:161835. doi:10.1016/j.scitotenv.2023.161835 36731559

[3] Scopetani C, Pellinen J, Selonen S. 2024. Phthalates and other organic chemicals in agricultural soils after use of different types of conventional and biodegradable plastics. Environ Res 255:119177. doi:10.1016/j.envres.2024.119177 38788789

[4] Chen YS, Huang YH, Lü HX, Zhao HM, Xiang L, Li H, Mo CH, Li YW, Cai QY. 2024. Simultaneous biodegradation of polycyclic aromatic hydrocarbons and phthalates by bacterial consortium and its bioremediation for complex polluted soil and sewage sludge. Bioresour Technol 408:131161. doi:10.1016/j.biortech.2024.131161 39067710

[5] Long FY, Ren YQ, Ji YY, Li JL, Zhang HJ, Wu ZH, Gao R, Bi F, Liu ZY, Li H. 2024. Pollution characteristics, toxicological properties, and health risk assessment of phthalic acid esters in water, soil, and atmosphere. Atmosphere (Basel) 15:1071. doi:10.3390/atmos15091071

[6] Net S, Delmont A, Sempéré R, Paluselli A, Ouddane B. 2015. Reliable quantification of phthalates in environmental matrices (air, water, sludge, sediment and soil): a review. Sci Total Environ 515–516:162–180. doi:10.1016/j.scitotenv.2015.02.013 25723871

[7] Huang YHZ, Chen KJ, Chen YH, Chen PP, Ge CL, Wang X, Huang C. 2024. Distribution of microplastics and phthalic acid esters during dry anaerobic digestion of food waste and potential microbial degradation analysis. Bioresour Technol 408:131221. doi:10.1016/j.biortech.2024.131221 39111396

[8] Qu Y, Chen J, Russel M, Huang W, Bingke Y, lei W, Zhang D, Blaszczak-Boxe C. 2024. Optimizing concentration and interaction mechanism of Demodesmus sp. and Achromobacter pulmonis sp. consortium to evaluate their potential for dibutyl phthalate removal from synthetic wastewater. Bioresour Technol 395:130372. doi:10.1016/j.biortech.2024.130372 38278454

[9] Sahoo TP, Kumar MA. 2023. Remediation of phthalate acid esters from contaminated environment-Insights on the bioremedial approaches and future perspectives. Heliyon 9:e14945. doi:10.1016/j.heliyon.2023.e14945 37025882 PMC10070671

[10] Hara H, Stewart GR, Mohn WW. 2010. Involvement of a novel ABC transporter and monoalkyl phthalate ester hydrolase in phthalate ester catabolism by Rhodococcus jostii RHA1. Appl Environ Microbiol 76:1516–1523. doi:10.1128/AEM.02621-09 20038686 PMC2832387

[11] Mutanda I, Sun JZ, Jiang JX, Zhu DC. 2022. Bacterial membrane transporter systems for aromatic compounds: regulation, engineering, and biotechnological applications. Biotechnol Adv 59:107952. doi:10.1016/j.biotechadv.2022.107952 35398204

[12] Qiao P, Ying TT, Gu MJ, Zhu JH, Mei CY, Hu T, Liu TF, Wang HX, Zhong WH. 2024. Assimilation of phthalate esters in bacteria. Appl Microbiol Biotechnol 108:276. doi:10.1007/s00253-024-13105-6 38536521 PMC10973024

[13] Zhao LL, Wei JY, Pan X, Jie Y, Zhu BQ, Zhao HF, Zhang BL. 2021. Critical analysis of peptidoglycan structure of Lactobacillus acidophilus for phthalate removal. Chemosphere 282:130982. doi:10.1016/j.chemosphere.2021.130982 34111639

[14] Zhang Y, Shi HT, Gu JD, Jiao YQ, Han SY, Akindolie MS, Wang YF, Zhang L, Tao Y. 2020. Anthraquinone-2,6-disulfonate enhanced biodegradation of dibutyl phthalate: reducing membrane damage and oxidative stress in bacterial degradation. Bioresour Technol 302:122845. doi:10.1016/j.biortech.2020.122845 32000129

[15] Meng Q, Liang YX, Xu YM, Li SY, Huang HY, Xu YY, Cao FF, Yin JH, Zhu TH, Gao HC, Yu ZL. 2025. A novel FadL family outer membrane transporter is involved in the uptake of polycyclic aromatic hydrocarbons. Appl Environ Microbiol 91. doi:10.1128/aem.00827-24 PMC11837497 39853126

[16] Liu J, Chen S, Zhao B, Li G, Ma T. 2022. A novel FadL homolog, AltL, mediates transport of long-chain alkanes and fatty acids in acinetobacter venetianus RAG-1. Appl Environ Microbiol 88:e01294–22. doi:10.1128/aem.01294-22 36169310 PMC9599521

[17] Jiang DH, Zhao Y, Wang XP, Fan JP, Heng J, Liu XH, Feng W, Kang XS, Huang B, Liu JF, Zhang XJC. 2013. Structure of the YajR transporter suggests a transport mechanism based on the conserved motif A. Proc Natl Acad Sci USA 110:14664–14669. doi:10.1073/pnas.1308127110 23950222 PMC3767500

[18] Wu HH, Symersky J, Lu M. 2020. Structure and mechanism of a redesigned multidrug transporter from the major facilitator superfamily. Sci Rep 10:3949. doi:10.1038/s41598-020-60332-8 32127561 PMC7054563

[19] Deng FR, Zhao L, Wei P, Mai EH, Chen MC, Yang HX, Mu PQ, Wu J, Wen JK, Deng YQ. 2024. Role and mechanism of the outer membrane porin LamB in T-2 mycotoxin-mediated extensive drug resistance in Escherichia coli. J Hazard Mater 480:136437. doi:10.1016/j.jhazmat.2024.136437 39541888

[20] Pahil KS, Gilman MSA, Baidin V, Clairfeuille T, Mattei P, Bieniossek C, Dey F, Muri D, Baettig R, Lobritz M, Bradley K, Kruse AC, Kahne D. 2024. A new antibiotic traps lipopolysaccharide in its intermembrane transporter. Nature 625:572–577. doi:10.1038/s41586-023-06799-7 38172635 PMC10794137

[21] Clifton BE, Alcolombri U, Uechi G-I, Jackson CJ, Laurino P. 2024. The ultra-high affinity transport proteins of ubiquitous marine bacteria. Nature 634:721–728. doi:10.1038/s41586-024-07924-w 39261732 PMC11485210

[22] Jabara N, Pollet R. 2024. Abstract 1686 characterizing the role of TonB7 in bacteroides thetaiotaomicron. J Biol Chem 300:105858. doi:10.1016/j.jbc.2024.105858

[23] Heidarinia H, Tajbakhsh E, Rostamian M, Momtaz H. 2023. Epitope mapping of Acinetobacter baumannii outer membrane protein W (OmpW) and laboratory study of an OmpW-derivative peptide. Heliyon 9:e18614. doi:10.1016/j.heliyon.2023.e18614 37560650 PMC10407128

[24] Delgado KN, Caimano MJ, Orbe IC, Vicente CF, La Vake CJ, Grassmann AA, Moody MA, Radolf JD, Hawley KL. 2024. Immunodominant extracellular loops of Treponema pallidum FadL outer membrane proteins elicit antibodies with opsonic and growth-inhibitory activities. PLoS Pathog 20:e1012443. doi:10.1371/journal.ppat.1012443 39715273 PMC11761103

[25] Drew D, North RA, Nagarathinam K, Tanabe M. 2021. Structures and general transport mechanisms by the Major Facilitator Superfamily (MFS). Chem Rev 121:5289–5335. doi:10.1021/acs.chemrev.0c00983 33886296 PMC8154325

[26] Sajid A, Rahman H, Ambudkar SV. 2023. Advances in the structure, mechanism and targeting of chemoresistance-linked ABC transporters. Nat Rev Cancer 23:762–779. doi:10.1038/s41568-023-00612-3 37714963

[27] Quistgaard EM, Löw C, Guettou F, Nordlund P. 2016. Understanding transport by the major facilitator superfamily (MFS): structures pave the way. Nat Rev Mol Cell Biol 17:123–132. doi:10.1038/nrm.2015.25 26758938

[28] Mori K, Niinuma K, Fujita M, Kamimura N, Masai E. 2018. DdvK, a novel major facilitator superfamily transporter essential for 5,5’-dehydrodivanillate uptake by Sphingobium sp. strain SYK-6. Appl Environ Microbiol 84:e01314-18. doi:10.1128/AEM.01314-18 30120118 PMC6182893

[29] Pernstich C, Senior L, MacInnes KA, Forsaith M, Curnow P. 2014. Expression, purification and reconstitution of the 4-hydroxybenzoate transporter PcaK from Acinetobacter sp. ADP1. Protein Expr Purif 101:68–75. doi:10.1016/j.pep.2014.05.011 24907408 PMC4148202

[30] Chaudhry MT, Huang Y, Shen XH, Poetsch A, Jiang CY, Liu SJ. 2007. Genome-wide investigation of aromatic acid transporters in Corynebacterium glutamicum. Microbiology (Reading, Engl) 153:857–865. doi:10.1099/mic.0.2006/002501-0 17322206

[31] Mori K, Kamimura N, Masai E. 2018. Identification of the protocatechuate transporter gene in Sphingobium sp. strain SYK-6 and effects of overexpression on production of a value-added metabolite. Appl Microbiol Biotechnol 102:4807–4816. doi:10.1007/s00253-018-8988-3 29675799

[32] Choudhary A, Purohit H, Phale PS. 2017. Benzoate transport in Pseudomonas putida CSV86. FEMS Microbiol Lett 364:fnx118. doi:10.1093/femsle/fnx118 28591829

[33] Cillingová A, Zeman I, Tóth R, Neboháčová M, Dunčková I, Hölcová M, Jakúbková M, Gérecová G, Pryszcz LP, Tomáška Ľ, Gabaldón T, Gácser A, Nosek J. 2017. Eukaryotic transporters for hydroxyderivatives of benzoic acid. Sci Rep 7:8998. doi:10.1038/s41598-017-09408-6 28827635 PMC5566891

[34] D’Arrigo I, Cardoso JGR, Rennig M, Sonnenschein N, Herrgård MJ, Long KS. 2019. Analysis of Pseudomonas putida growth on non-trivial carbon sources using transcriptomics and genome-scale modelling. Environ Microbiol Rep 11:87–97. doi:10.1111/1758-2229.12704 30298597

[35] Xu Y, Chen B, Chao HJ, Zhou NY. 2013. mhpT encodes an active transporter involved in 3-(3-hydroxyphenyl)propionate catabolism by Escherichia coli K-12. Appl Environ Microbiol 79:6362–6368. doi:10.1128/AEM.02110-13 23934492 PMC3811207

[36] Pardo I, Jha RK, Bermel RE, Bratti F, Gaddis M, McIntyre E, Michener W, Neidle EL, Dale T, Beckham GT, Johnson CW. 2020. Gene amplification, laboratory evolution, and biosensor screening reveal MucK as a terephthalic acid transporter in Acinetobacter baylyi ADP1. Metab Eng 62:260–274. doi:10.1016/j.ymben.2020.09.009 32979486

[37] Wada A, Prates ÉT, Hirano R, Werner AZ, Kamimura N, Jacobson DA, Beckham GT, Masai E. 2021. Characterization of aromatic acid/proton symporters in Pseudomonas putida KT2440 toward efficient microbial conversion of lignin-related aromatics. Metab Eng 64:167–179. doi:10.1016/j.ymben.2021.01.013 33549838

[38] Nijland M, Lefebvre SN, Thangaratnarajah C, Slotboom DJ. 2024. Bidirectional ATP-driven transport of cobalamin by the mycobacterial ABC transporter BacA. Nat Commun 15:2626. doi:10.1038/s41467-024-46917-1 38521790 PMC10960864

[39] Li C-Y, Mausz MA, Murphy A, Zhang N, Chen X-L, Wang S-Y, Gao C, Aguilo-Ferretjans MM, Silvano E, Lidbury IDEA, Fu H-H, Todd JD, Chen Y, Zhang Y-Z. 2023. Ubiquitous occurrence of a dimethylsulfoniopropionate ABC transporter in abundant marine bacteria. ISME J 17:579–587. doi:10.1038/s41396-023-01375-3 36707613 PMC10030565

[40] Liu TF, Li J, Qiu LQ, Zhang FM, Linhardt RJ, Zhong WH. 2020. Combined genomic and transcriptomic analysis of the dibutyl phthalate metabolic pathway in Arthrobacter sp. ZJUTW. Biotechnol Bioeng 117:3712–3726. doi:10.1002/bit.27524 32740909

[41] Hu T, Yang C, Hou ZY, Liu TF, Mei XT, Zheng LB, Zhong WH. 2022. Phthalate esters metabolic strain Gordonia sp. GZ-YC7, a potential soil degrader for high concentration Di-(2-ethylhexyl) phthalate. Microorganisms 10:641. doi:10.3390/microorganisms10030641 35336217 PMC8955600

[42] Hou ZY, Pan HJ, Gu MJ, Chen XW, Ying TT, Qiao P, Cao JW, Wang HX, Hu T, Zheng LB, Zhong WH. 2024. Simultaneously degradation of various phthalate esters by Rhodococcus sp. AH-ZY2: strain, omics and enzymatic study. J Hazard Mater 474:134776. doi:10.1016/j.jhazmat.2024.134776 38852255

[43] Sandhu G, Khan A, Trivedi PK. 2025. Transport channels enabling uptake, translocation and detoxification of arsenic in plants. Plant Physiol Biochem 225:109994. doi:10.1016/j.plaphy.2025.109994 40408928

[44] Wu X, Ren J, Wang J, Koffas MAG, Zha J. 2024. A major facilitator superfamily transporter MdtH in Escherichia coli is involved in anthocyanin biosynthesis and secretion. Appl Environ Microbiol 90:e0207923. doi:10.1128/aem.02079-23 38349148 PMC10952383

[45] Smirnova I, Kasho V, Choe JY, Altenbach C, Hubbell WL, Kaback HR. 2007. Sugar binding induces an outward facing conformation of LacY. Proc Natl Acad Sci USA 104:16504–16509. doi:10.1073/pnas.0708258104 17925435 PMC2034228

[46] Krishnamurthy H, Piscitelli CL, Gouaux E. 2009. Unlocking the molecular secrets of sodium-coupled transporters. Nature 459:347–355. doi:10.1038/nature08143 19458710 PMC6821466

[47] Wandel S, Schurmann A, Becker W, Summers ScottA, Shanahan MichaelF, Joost HansG. 1995. Mutation of two conserved arginine residues in the glucose transporter GLUT4 supresses transport activity, but not glucose-inhibitable binding of inhibitory ligands. N-S Arch Pharmacol 353:36–41. doi:10.1007/BF00168913 8750914

[48] Yan HC, Huang WY, Yan CY, Gong X, Jiang S, Zhao Y, Wang JW, Shi YG. 2013. Structure and mechanism of a nitrate transporter. Cell Rep 3:716–723. doi:10.1016/j.celrep.2013.03.007 23523348

[49] Fukuda M, Takeda H, Kato HE, Doki S, Ito K, Maturana AD, Ishitani R, Nureki O. 2015. Structural basis for dynamic mechanism of nitrate/nitrite antiport by NarK. Nat Commun 6:7097. doi:10.1038/ncomms8097 25959928 PMC4432589

[50] Micciulla JL, Shor LM, Gage DJ. 2024. Enhanced transport of bacteria along root systems by protists can impact plant health. Appl Environ Microbiol 90:e0201123. doi:10.1128/aem.02011-23 38534145 PMC11022564

[51] Xu S, Li Q, Li Y, Zhang Y, Li Q, Ji L, Cheng H. 2025. Synergistic effect of transporter and pathway engineering on the key performance indicators of erythritol synthesis by the yeast Yarrowia lipolytica. Appl Environ Microbiol 91:e0006125. doi:10.1128/aem.00061-25 40135906 PMC12016529

[52] Marreddy RKR, Pinto JPC, Wolters JC, Geertsma ER, Fusetti F, Permentier HP, Kuipers OP, Kok J, Poolman B. 2011. The response of Lactococcus lactis to membrane protein production. PLoS One 6:e24060. doi:10.1371/journal.pone.0024060 21904605 PMC3164122

[53] Liu TF, Ning LX, Mei CY, Li S, Zheng LB, Qiao P, Wang HX, Hu T, Zhong WH. 2023. Synthetic bacterial consortia enhanced the degradation of mixed priority phthalate ester pollutants. Environ Res 235:116666. doi:10.1016/j.envres.2023.116666 37453507

[54] Ferreira ID, Morita DM. 2012. Ex-situ bioremediation of Brazilian soil contaminated with plasticizers process wastes. Braz J Chem Eng 29:77–86. doi:10.1590/S0104-66322012000100009

[55] Guo DM, Ying WU. 2011. Detemination of phthalic acid esters of soil in south of Xinjiang cotton fields. Arid Environ Monit 25:76–79. https://api.semanticscholar.org/CorpusID:130508078.

[56] Morris GM, Huey R, Olson AJ. 2008. Using AutoDock for ligand-receptor docking. Curr Protoc Bioinformatics Chapter 8.14. doi:10.1002/0471250953.bi0814s24 19085980

