# Supplementary material for: Multiple novel membrane proteins involve phthalate ester degradation in Rhodococcus sp. AH-ZY2
Source: Appl Environ Microbiol. 2026 Apr 29;92(5):e02526-25. doi: 10.1128/aem.02526-25 (PMC13188901; doi:10.1128/aem.02526-25)
Supplement: Supplemental material — Fig. S1 to S9; Tables S1 to S5. [file aem.02526-25-s0001.docx]

**Table S1 MFS family transporters for transport of aromatic compounds**

| Aromatic compound | Structural formula | Name | MFS family | Strains | References |
| --- | --- | --- | --- | --- | --- |
| 5,5′-dehydrodivanillate | 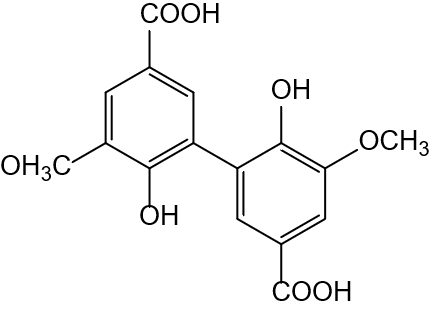 | DdvK | AAHS | *Sphingobium* sp. SYK-6 | (1) |
| Protocatechuic acid | 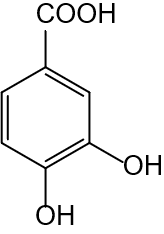 | PcaK | AAHS | *Acinetobacter baylyi* ADP1 | (2) |
|  |  | PcaK | AAHS | *Corynebacterium glutamicum* | (3) |
|  |  | PcaK | AAHS | *Sphingobium* SYK-6 | (4) |
| Vanillate & protocatechuic acid | 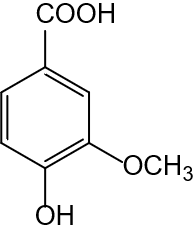＆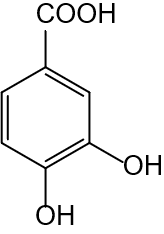 | VanK | AAHS | *P*. *putida* KT2440 | (5) |
| Benzoate | 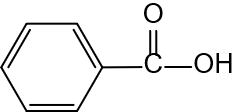 | BenK | AAHS | *Pseudomonas putida* CSV86 | (6) |
|  |  | BenE | BenE | *Pseudomonas putida* CSV86 | (6) |
|  |  | Hbt1/Hbt2 | AAHS | *Candida parapsilosis* | (7) |
| Ferulate | 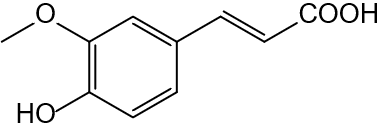 | PcaT | MHS | *P*. *putida* KT2440 | (8) |
| 3-(3- hydroxyphenyl) propionate | 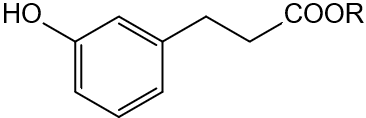 | MhpT | AAHS | *Escherichia coli* K-12 | (9) |
| Terephthalate | 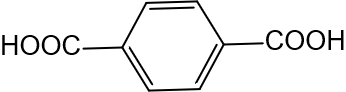 | Muck | AAHS | *Acinetobacter baylyi ADP1* | (10) |

**Table S1 (Continued) MFS family transporters for transport of aromatic compounds**

| Aromatic compound | Structural formula | Transporter Name | MFS family | Strains | References |
| --- | --- | --- | --- | --- | --- |
| 4-Coumarate | 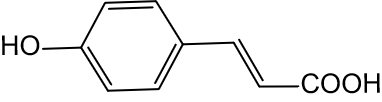 | Hcnk | AAHS | *P*. *putida* KT2440 | (5) |
| Benzalkonium bromide | 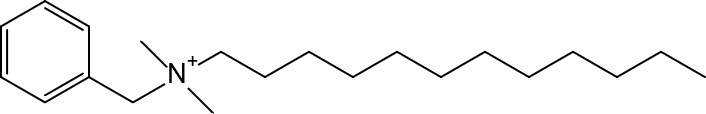 | EmrD | DHA1 | *E*. *coli* | (11) |
| Chloramphenicol | 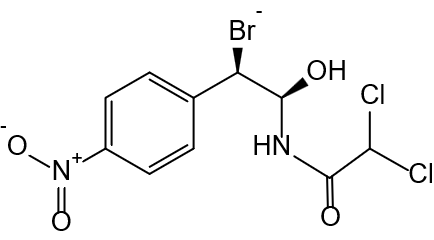 | YajR | DHA1 | *E*. *coli* | (11) |
|  |  | MdfA | DHA1 | *E*. *coli* | (12) |
| Phthalate esters | 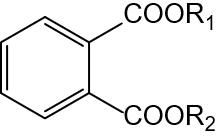 | AH-ZY2-*0620* | NNP | *Rhodococcus* sp. AH-ZY2 | This article |
|  |  | AH-ZY2-*3572* | MHS | *Rhodococcus* sp. AH-ZY2 | This article |
|  |  | AH-ZY2-*5299* | UMF22 | *Rhodococcus* sp. AH-ZY2 | This article |

Note: AH-ZY2-0620 (UhpC). AH-ZY2-3572 (ProP), AH-ZY2-5299 (TraP). DHA1 Family: The Drug:H+ Antiporter-1 (12 Spanner) (DHA1) Family. AAHS Family: The Aromatic Acid:H+ Symporter (AAHS) Family. UMF22 Family: The Unidentifed Major Facilitator-22 (UMF22) Family. MHS Family: The Metabolite:H+ Symporter (MHS) Family. NNP Family: The Nitrate/Nitrite Porter (NNP) family. BenE Family: Benzoate-H+ symporter. Red represents the transporters studied in this article.

**Table S2 Substrate spectrum and efficiency of PAEs degraded by *Glutamicibacter* sp. ZJUTW, *Gordonia* sp. GZ-YC7 and *Rhodococcus* sp. AH-ZY2**

| Strains | Substrate spectrum | Degrading efficiency | Reference |
| --- | --- | --- | --- |
| ZJUTW | DBP^#^, DMP, DEP | 400 mg/L DBP, 93.4% degraded in 14 h | (13) |
| GZ-YC7 | DEHP^#^, DEP, DBP, DPrP, BBP, DnOP, DiNP | 500 mg/L DEHP, 100% degraded in 24 h | (14) |
| AH-ZY2 | DnOP^#^, DMP, DEP, DBP, DPrP, BBP, DEHP, DiNP | 500 mg/L DnOP, 100% degraded in 16 h | (15) |

^#^: The substrate with the fastest degradation efficiency by this strain. DMP (Dimethyl phthalate), DEP (Diethyl phthalate), DPrP (Dipropyl phthalate), DBP (Dibutyl phthalate), BBP (Benzyl butyl phthalate), DEHP (Bis(2-ethylhexyl) phthalate), DnOP (Di-n-octyl-phthalate), DiNP (Diisononyl phthalate)

**Table S3 Summary of predicted esterase genes in ZJUTW, GZ-YC7, AH-ZY2 and functional verification in *E. coil* BL21(DE3)**

| **Strains** | **Gene Numbers** | **Referenced esterase** | **Referenced Source of esterase** | **Sequence Similarity（%）** | **Function**  **(Yes or No)** | **Substrate spectrum** | **Experimental verification of esterase type** |
| --- | --- | --- | --- | --- | --- | --- | --- |
| ZJUTW | 0024 | PehA | *Gordonia* sp. YC-JH1 | 25.00 | Yes | DMP, DEP, DBP | I |
|  | 0047 | CarEW | Bacillus sp. K91 | 36.39 | Yes | DMP，DEP，DPrP，DBP，BBP，DEHP，DnOP，DiNP | III |
| GZ-YC7 | 4058 | QHH19548.1 | *Bacillus subtilis* | 20.50 | No | / | / |
|  | 3243 | QHH19548.1 | *Bacillus subtilis* | 24.00 | No | / | / |
|  | 1667 | QHH19548.1 | *Bacillus subtilis* | 23.50 | No | / | / |
|  | 2053 | QHH19548.1 | *Bacillus subtilis* | 21.30 | No | / | / |
|  | 3151 | QHH19548.1 | *Bacillus subtilis* | 18.00 | No | / | / |
|  | 4329 | QHH19548.1 | *Bacillus subtilis* | 19.60 | No | / | / |
|  | 1189 | MehpH | *Gordonia* sp. P8219 | 98.94 | Yes | MBP, MEHP | II |
|  | 0714 | CarEW | *Bacillus* sp. K91 | 33.80 | Yes | DPrP, DBP, BBP | I |
| AH-ZY2 | 5411 | EstS1 | *Sulfobacillus acidophilus* DSM 10332 | 26.98 | Yes | DMP，DEP，DPrP，DBP，BBP | I |
|  | 2161 | EstS1 | *Sulfobacillus acidophilus* DSM 10332 | 36.34 | Yes | DBP | I |
|  | 1835 | EstSP1 | *Sphingomonas glacialis* PAMC 26605 | 24.71 | Yes | DMP，DEP，DPrP，DBP，BBP | I |
|  | 4329 | DphB | Uncultured bacterium | 25.92 | Yes | DMP，DEP，DPrP，DBP，BBP | I |
|  | 0740 | Hyd | *Rhodococcus* sp.2G | 70.40 | No | / | / |
|  | 4158 | EstB | *Sphingobium* sp. SM42 | 24.69 | Yes | DBP | I |
|  | 3693 | EstB | *Sphingobium* sp. SM42 | 21.06 | Yes | DBP | I |
|  | 3963 | Carboxylesterase | *Rhodococcus* sp. LW-XY12 | 69.31 | Yes | DMP，DEP，DPrP，DBP，BBP | I |
|  | 4547 | Carboxylesterase | *Rhodococcus* sp. LW-XY12 | 41.78 | Yes | DMP，DEP，DPrP，DBP，BBP | I |
|  | 2121 | EstG | *Sphingobium* sp. SM42 | 24.80 | Yes | DMP，DEP，DPrP，DBP，BBP | I |
|  | 5294 | MehpH | *Gordonia* sp. P8219 | 100.00 | Yes | MBP, MEHP | II |
|  | 5359 | Carboxylesterase | *Rhodococcus* sp. LW-XY12 | 57.88 | Yes | DMP，DEP，DPrP，DBP，BBP，DEHP，DnOP，DiNP | III |

Note: DMP (Dimethyl phthalate), DEP (Diethyl phthalate), DPrP (Dipropyl phthalate), DBP (Dibutyl phthalate), BBP (Benzyl butyl phthalate), DEHP (Bis(2-ethylhexyl) phthalate), DnOP (Di-n-octyl-phthalate), DiNP (Diisononyl phthalate)

**Table S4 The MFS annotated in the genomes of the ZJUTW, GZ-YC7 and AH-ZY2 may be related to the transport of PAEs.**

| Strains | Serial number | Gene number | Log_2_F(c) | Function Description |
| --- | --- | --- | --- | --- |
| ZJUTW | 1 | *2727* | 8.2445 | MFS transporter |
|  | 2 | *3361* | 6.2315 | MFS transporter |
|  | 3 | *3334* | 5.697 | MFS transporter |
|  | 4-15 | *1132-…* | 5.5792-2.1876 | MFS transporter |
|  | 16 | *0002* | 2.1756 | MFS transporter  MFS transporter |
|  | 17-31 | *0433-…* | 2.1706-0.5612 | MFS transporter |
| GZ-YC7 | 1 | *1489* | 4.9199 | MFS transporter |
|  | 2 | *2515* | 4.2726 | MFS transporter |
|  | 3 | *3965* | 4.0487 | MFS transporter |
|  | 4 | *2203* | 3.4531 | MFS transporter |
|  | 5 | *0616* | 1.2536 | MFS transporter |
|  | 6-12 | *4117...* | 0.9321-0.5008 | MFS transporter |
| AH-ZY2 | 1 | *3662* | 9.3226 | MFS transporter |
|  | 2 | *5299* | 5.1941 | MFS transporter |
|  | 3-6 | *4495-…* | 4.1496-2.8714 | MFS transporter |
|  | 7 | *3107* | 2.7436 | MFS transporter |
|  | 8 | *4876* | 2.3605 | MFS transporter |
|  | 9-27 | *2875-…* | 2.0685-0.5417 | MFS transporter |

Note：ZJUTW used DBP as the substrate to detect the transcription level (log_2_f(c)>0.5) of the transporter, GZ-YC7 used DEHP as the substrate to detect the transcription level (log_2_f(c)>0.5) of the transporter, and AH-ZY2 used DnOP as the substrate to detect the transcription level (log_2_f(c)>0.5) of the transporter.

**Table S5** Primers sequences of gene involved in this articlestudy

| Primers Name | Primer Sequences |
| --- | --- |
| 0620-UF | AGCTCGGTACCCGGGGATCCcgtccactgtccgtcctgc |
| 0620-UR | ctgaccccgtcccggtgcgtgtgggagcctatacctgggc |
| 0620-DF | ccaggtataggctcccacacgcaccgggacggggtcag |
| 0620-DR | CGACGGCCAGTGCCAAGCTTttcgagctcgcgcagagc |
| 3572-UF | AGCTCGGTACCCGGGGATCCgtgatgctggcgttgtgggg |
| 3572-UR | ccggagagggggttcggaggcagatgaccgggtcggacc |
| 3572-DF | ggtccgacccggtcatctgcctccgaaccccctctccgg |
| 3572-DR | CGACGGCCAGTGCCAAGCTTgttcctgaacaagcggtcggtg |
| 4497-UF | AGCTCGGTACCCGGGGATCCtacggggagctcatcgacgg |
| 4497-UR | gttgacgatgggttctcctggaggggaagaggagg |
| 4497-DF | cctccaggagaacccatcgtcaacatcgtcgg |
| 4497-DR | CGACGGCCAGTGCCAAGCTTaggaagacggtcggctcg |
| 5299-UF | AGCTCGGTACCCGGGGATCCgtcgcctacgatgcggaccttc |
| 5299-UR | ggtctgtgtcggtgtgctttcccgtggt |
| 5299-DF | accacgggaaagcacaccgacacagaccccgaggct |
| 5299-DR | CGACGGCCAGTGCCAAGCTTaccgatcgctttgacacgg |
| 0620-complement F | CGACGGCCAGTGCCAAGCTTtcatttgcgggggaccagg |
| 0620-complement R | CGAGCTCGGTACCCGGGGATCCatgacggttcgggacatcg |
| 3572-complement F | AACGACGGCCAGTGCCAAGCTTtcaggccttcgacagcg |
| 3572-complement R | CGAGCTCGGTACCCGGGGATCCatgggcaacttcacggagtg |
| 4497-complement F | CGACGGCCAGTGCCAAGCTTGtcagggctgctcactggac |
| 4497-complement R | CTCGGTACCCGGGGATCCTTCatgcgcccctcccttc |
| 5299-complement F | ACGGCCAGTGCCAAGCTTtcagtcgacatctttgttggatctc |
| 5299-complement R | AGCTCGGTACCCGGGGATCcatggactccgtcaactatgacag |
| 0620-F | CGACGGCCAGTGCCAAGCTTtcatttgcgggggaccagg |
| 0620-R | CGAGCTCGGTACCCGGGGATCCatgacggttcgggacatcg |
| 3572-F | AACGACGGCCAGTGCCAAGCTTtcaggccttcgacagcg |
| 3572-R | CGAGCTCGGTACCCGGGGATCCatgggcaacttcacggagtg |
| 4497-F | CGACGGCCAGTGCCAAGCTTGtcagggctgctcactggac |
| 4497-R | CTCGGTACCCGGGGATCCTTCatgcgcccctcccttc |
| 5299-F | ACGGCCAGTGCCAAGCTTtcagtcgacatctttgttggatctc |
| 5229-R | AGCTCGGTACCCGGGGATCcatggactccgtcaactatgacag |

Note: The homologous arm of the plasmid is represented in uppercase, and the complementary part of the target gene sequence is represented in lowercase.


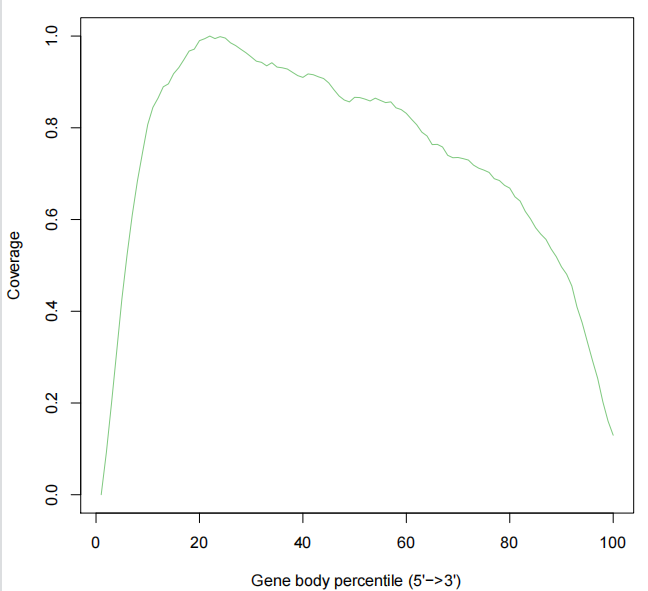


**Figure S1** Result of gene coverage analysis.

Note: The horizontal axis represents the percentage of the base length of a single gene relative to its total base length, where 0 indicates the 5' end of the gene and 100 indicates the 3' end. The vertical axis represents the total number of sequence reads mapped to the corresponding interval at each position along the horizontal axis across all genes. The figure displays the superimposed results of coverage for all genes, where the vertical coordinate of each point on the curve indicates the total number of sequences across all genes at that relative proportional position. The curve reflects whether the sequencing reads are evenly distributed along the genes. If a distinct peak appears near the left end, it indicates a significant 5' bias in the sequencing results. Conversely, a distinct peak near the right end indicates a significant 3' bias. In this figure, no significant peaks are observed at either end, indicating that the sequencing results are unbiased and relatively uniform.


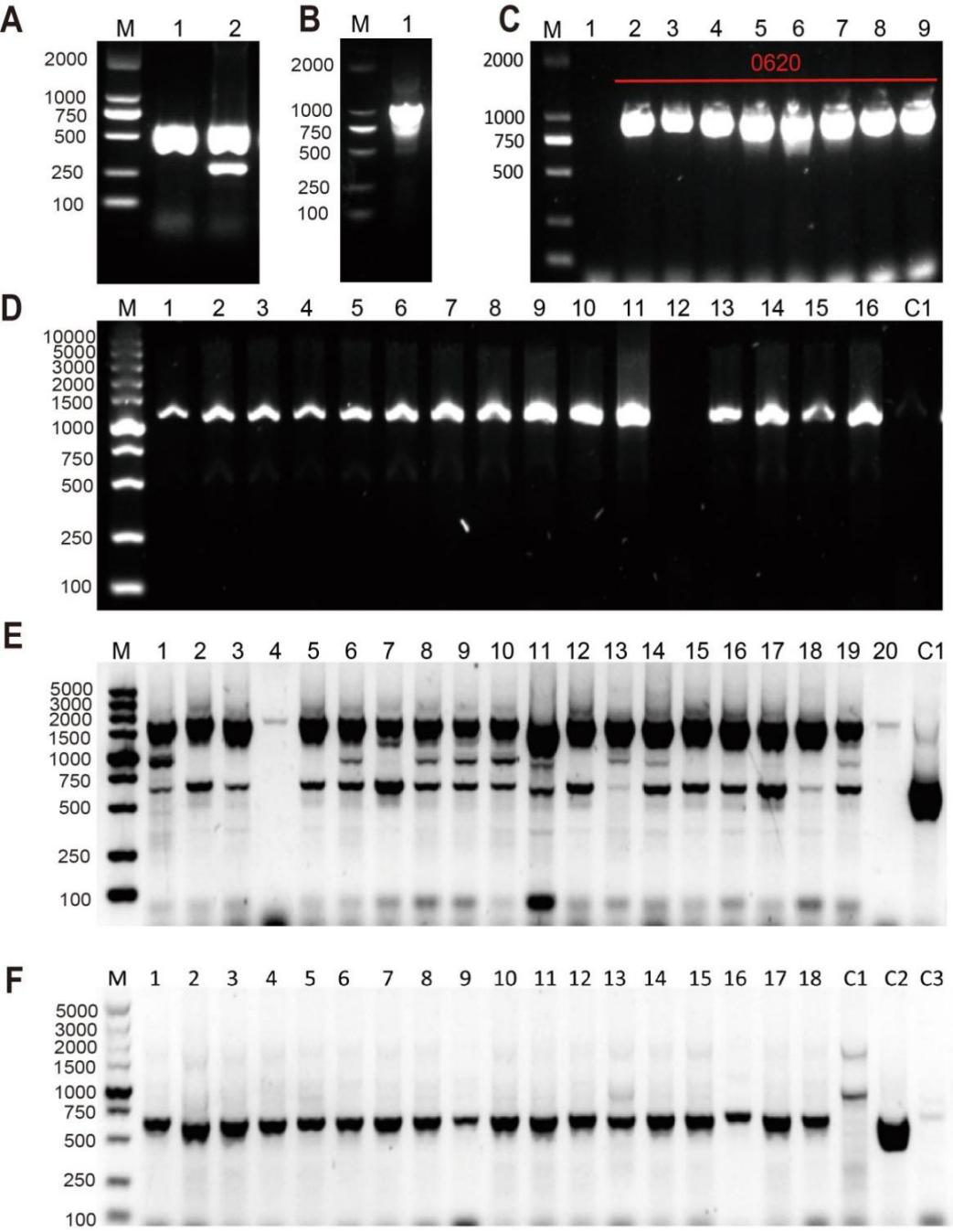


**Figure** **S2 Process validation of knocking out membrane protein 0620 gene.**

1, 0620U; 2, 0620D; M, Mark. **(A)**. 1: 0620U + 0620D; **(B)**. 1, H₂O; 2-9, PCR of the positive transformant bacterial liquid after the pK18*mobsacB*-0620 knockout vector was introduced into DH5α. **(C)**. 1-16: PCR of the positive transformant bacterial liquid after the pK18*mobsacB*-0620 knockout vector was introduced into WM3064. **(D)**. 1-19, the first screening transformants of the AH-ZY2-0620 knockout strain. 20, the wild strain. C1, plasmid. The small band represents 0620U + 0620D with a length of 687 bp. The large band represents 0620U + 0620 + 0620D with a length of 2243 bp. **(E)**. 1-8, AH-ZY2-0620 knockout transformants. C1, wild bacteria. C2, plasmid. C3, sterile water. The size of small band was 687bp, and the size of large band was 0620U+0620+ 0620D 2243bp **(F)**.


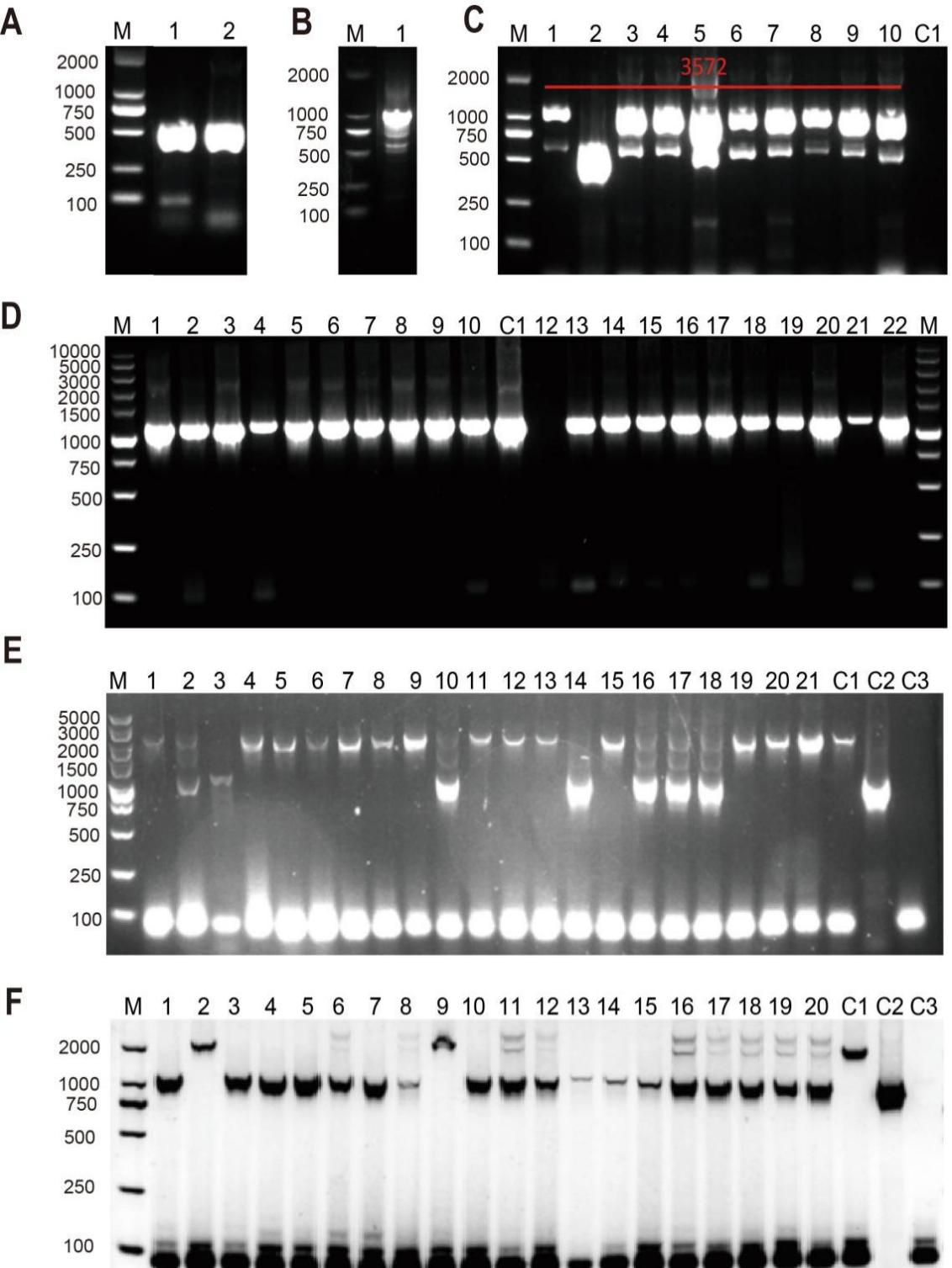


**Figure S3 Process validation of knocking out membrane protein 3572 gene.**

1, 3572U; 2, 3572D; M, Mark. **(A)**. 1: 3572U + 3572D; **(B)**. C1, H₂O; 1-10, PCR of the positive transformant bacterial liquid after the pK18*mobsacB*-3572 knockout vector was introduced into DH5α. **(C)**. 1-10, 13-22: PCR of the positive transformant bacterial liquid after the pK18*mobsacB*-3572 knockout vector was introduced into WM3064. C1, the pK18*mobsacB*-3572 plasmid; 12, water. **(D)**. 1-21, the first screening transformants of the AH-ZY2-3572 knockout strain. C1, the wild strain. C2, the plasmid; C3, water. The small band: 3572U + 3572D, with a length of 1117 bp. The large band: 3572U + 3572 + 3572D, with a length of 2404 bp. **(E)**. 1-20, AH-ZY2-3572 knockout transformants. C1, wild bacteria. C2, plasmid. C3, sterile water. Small band: 3572U + 3572D, 1117bp. Large strips: 3572U + 3572 + 3572D, 2404bp **(F)**.


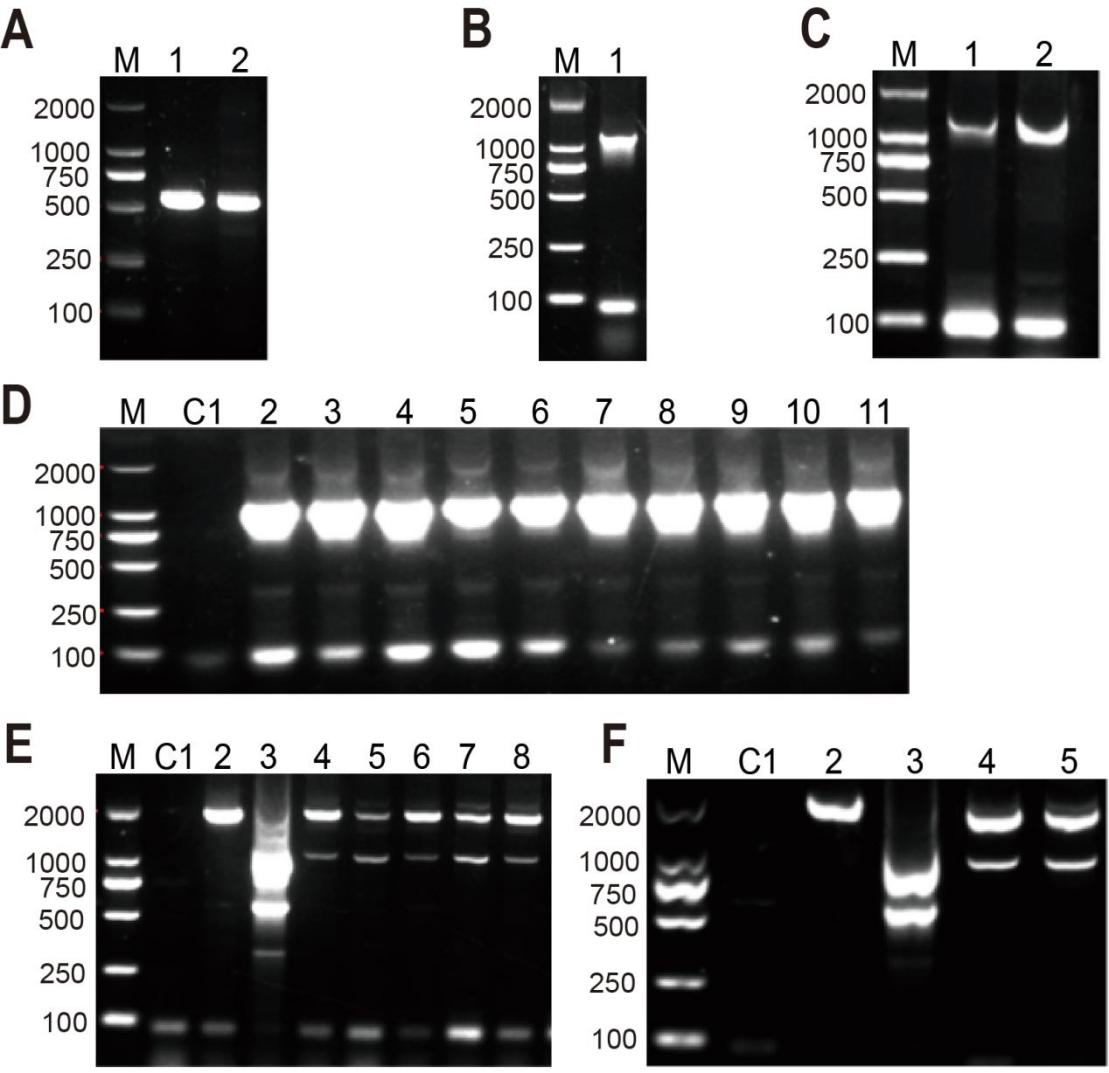


**Figure S4 Process validation of knocking out membrane protein 4497gene.**

1, 4497U; 2, 4497D; M, Mark. **(A)**. 1: 4497U + 4497D; **(B)**. 1-2, PCR of the positive transformant bacterial liquid after the pK18*mobsacB*-4497 knockout vector was introduced into DH5α. **(C)**. C1, H₂O. 3-11, PCR of the positive transformant bacterial liquid after the pK18*mobsacB*-4497 knockout vector was introduced into WM3064. 2, the pK18*mobsacB*-4497 plasmid. **(D)**. 3-8, the first screening transformants of the AH-ZY2-4497 knockout strain. C1, H₂O. 2, the wild strain. The small band: 4497U + 4497D, with a length of 1100 bp. The large band: 4497U + 4497 + 4497D, with a length of 2239 bp **(E)**. C1, water. 2, wild type. 3-5, AH-ZY2-4497 knockout transformants. Small band: 4497U + 4497D 1100bp Large band: 4497U + 4497 + 4497D 2239bp **(F)**.


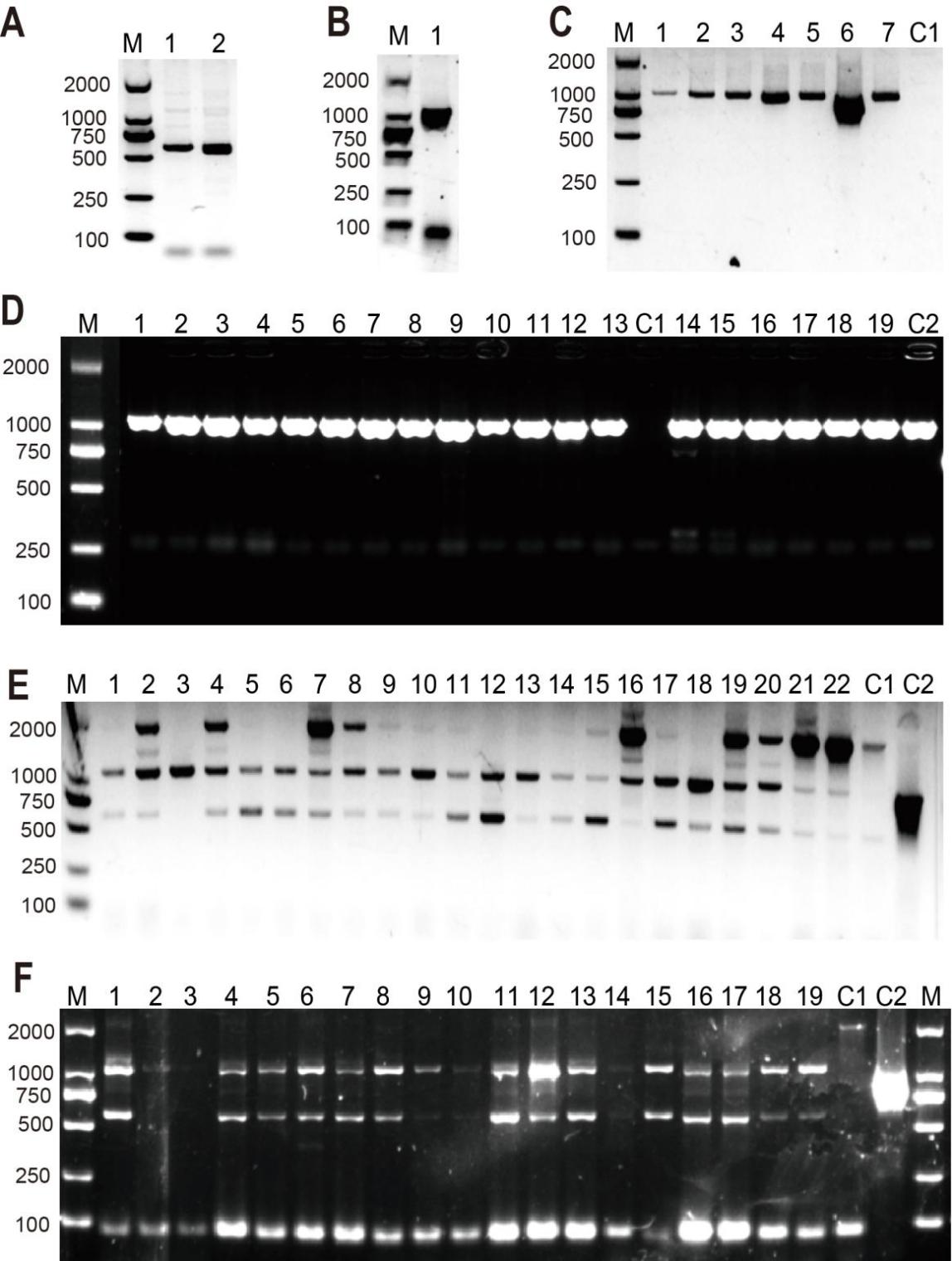


**Figure S5 Process validation of knocking out membrane protein 5299 gene.**

1, 5299U; 2, 5299D; M, Mark. **(A)**. 1: 5299U + 5299D; **(B)**. C1, H_2_O; 1-7, PCR of the positive transformant bacterial liquid after the pK18*mobsacB*-5299 knockout vector was introduced into DH5α. **(C)**. 1-13, 14-19: PCR of the positive transformant bacterial liquid after the pK18*mobsacB*-5299 knockout vector was introduced into WM3064. C1, water. C2, the pK18*mobsacB*-5299 plasmid. **(D)**. 1-22, PCR of the bacterial liquid of the first screening transformants of the AH-ZY2-5299 knockout strain. C1, the wild strain. C2, the plasmid. The small band: 5299U + 5299D, with a length of 1108 bp. The large band: 5299U + 5299 + 5299D, with a length of 2151 bp. **(E)**. 1-19, AH-ZY2-5299 knockout transformants. C1, wild bacteria. C2, plasmid. Small band: 5299U + 5299D, 1108bp. Large strips: 5299U + 5299 + 5299D, 2151bp **(F)**.


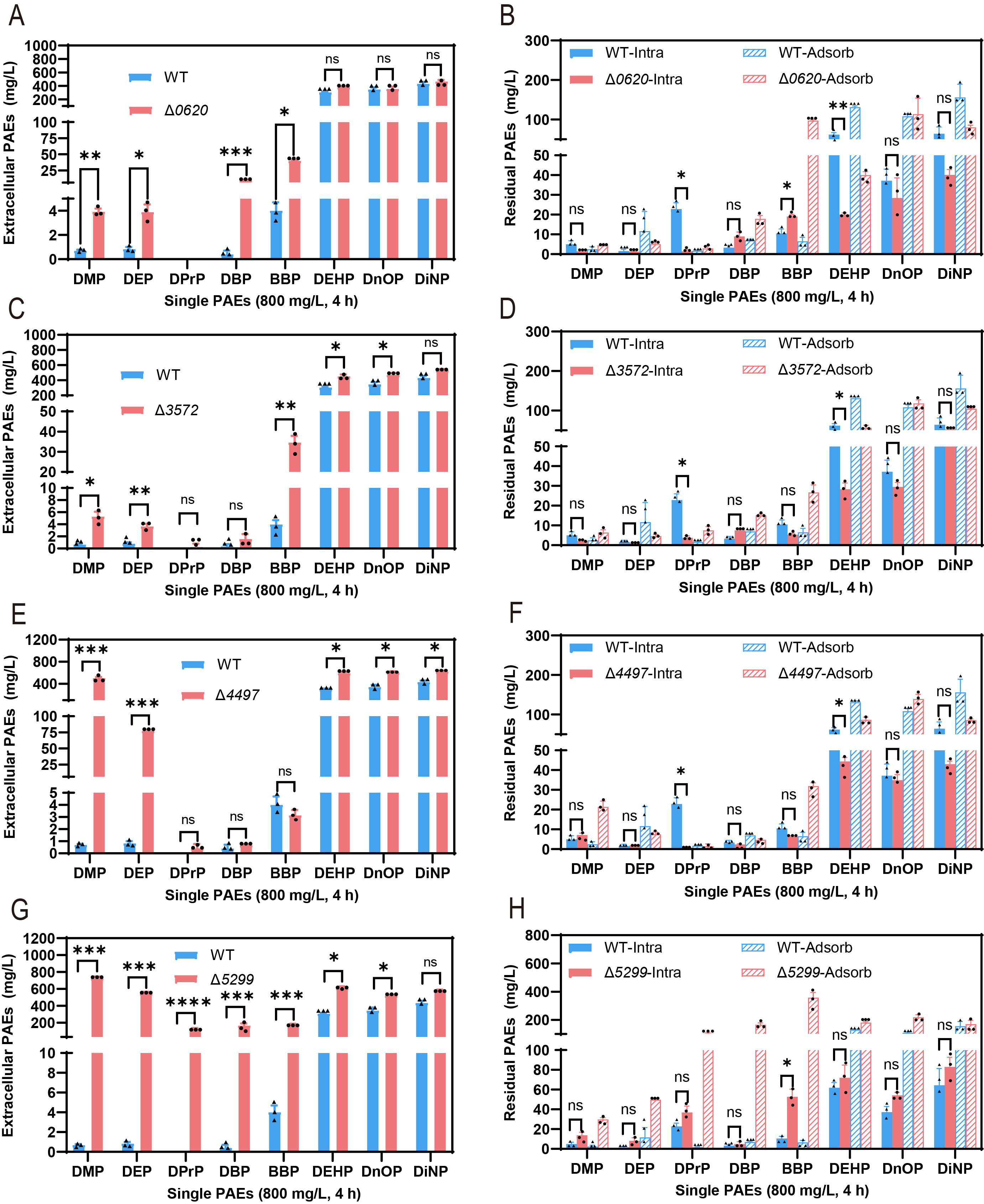


**Figure S6 Extracellular and intracellular PAEs concentration comparison between WT and knockout strains following exposure to eight individual PAEs (800 mg/L, 4 h).** Extracellular **(A)** and Intracellular **(B)** PAEs of strains WT and Δ*0620*. Extracellular- **(C)** and intracellular- **(D)** PAEs of strains WT and Δ*3572*. Extracellular- **(E)** and intracellular- **(F)** PAEs of strains WT and Δ*4497*. Extracellular- **(G)** and intracellular- **(H)** PAEs of strains WT and Δ*5299*. Bars represent mean ± SD from three independent experiments (n = 3). Note：*P* < 0.0001, ****. *P* < 0.001, ***. 0.001 < *P* < 0.01, **. 0.01<*P* < 0.05, *. *P* >0.1, ns.


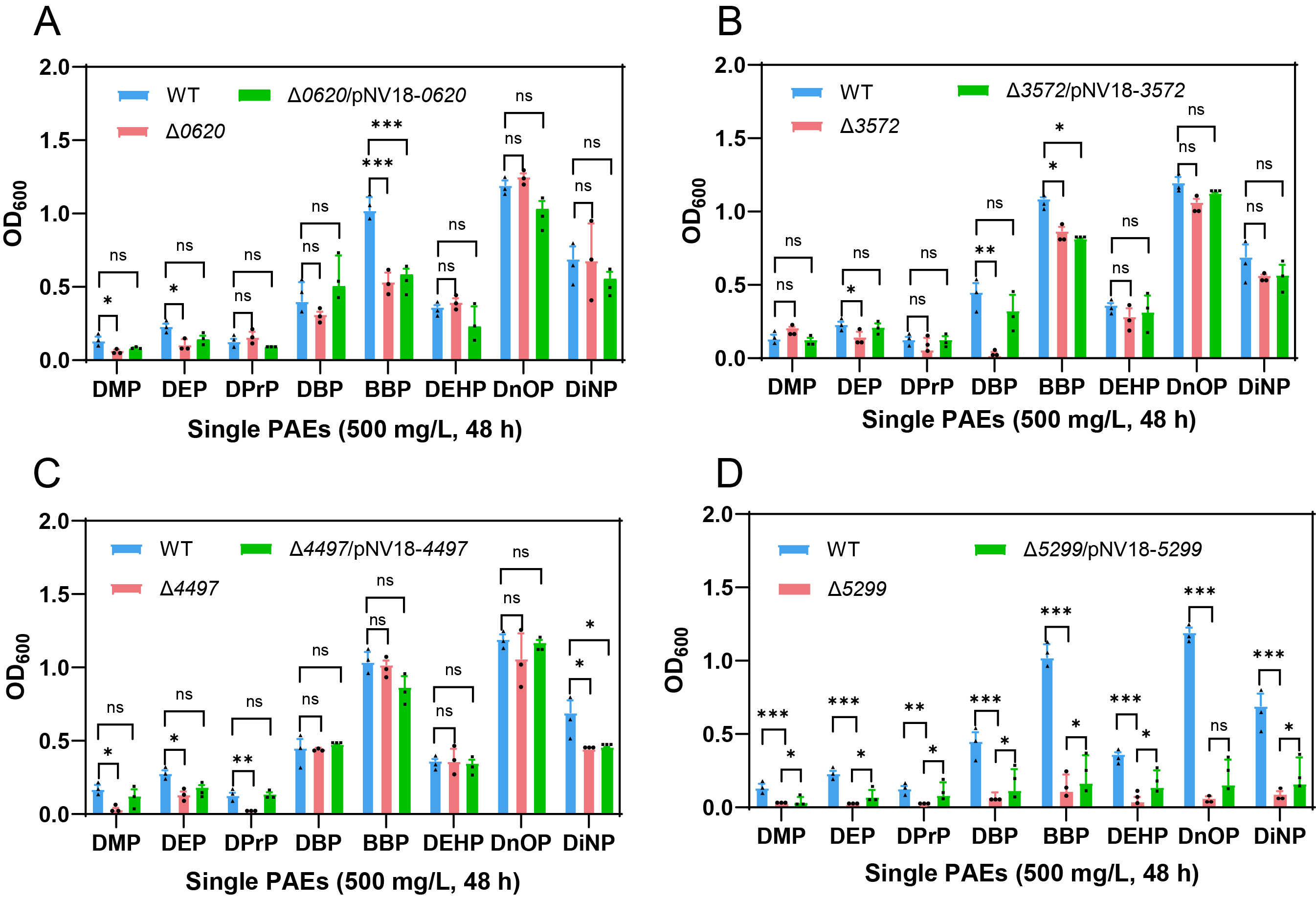


**Figure S7** **Growth assessment of WT and plasmid-complemented knock-out strains exposed to eight individual PAEs.** WT and plasmid complementation strainsΔ*0620*/pNV18-*0620* **(A)**, Δ*3572*/pNV18-*3572* **(B)**, Δ*4497*/pNV18-*4497* **(C)**, and Δ*5299*/pNV18-5299 **(D)** in BSM containing eight kinds of PAEs as the sole carbon source, respectively. Bars represent mean ± SD from three independent experiments (n = 3). Note：*P* < 0.001, ***. 0.001 < *P* < 0.01, **. 0.01<*P* < 0.05, *. *P* >0.1, ns.


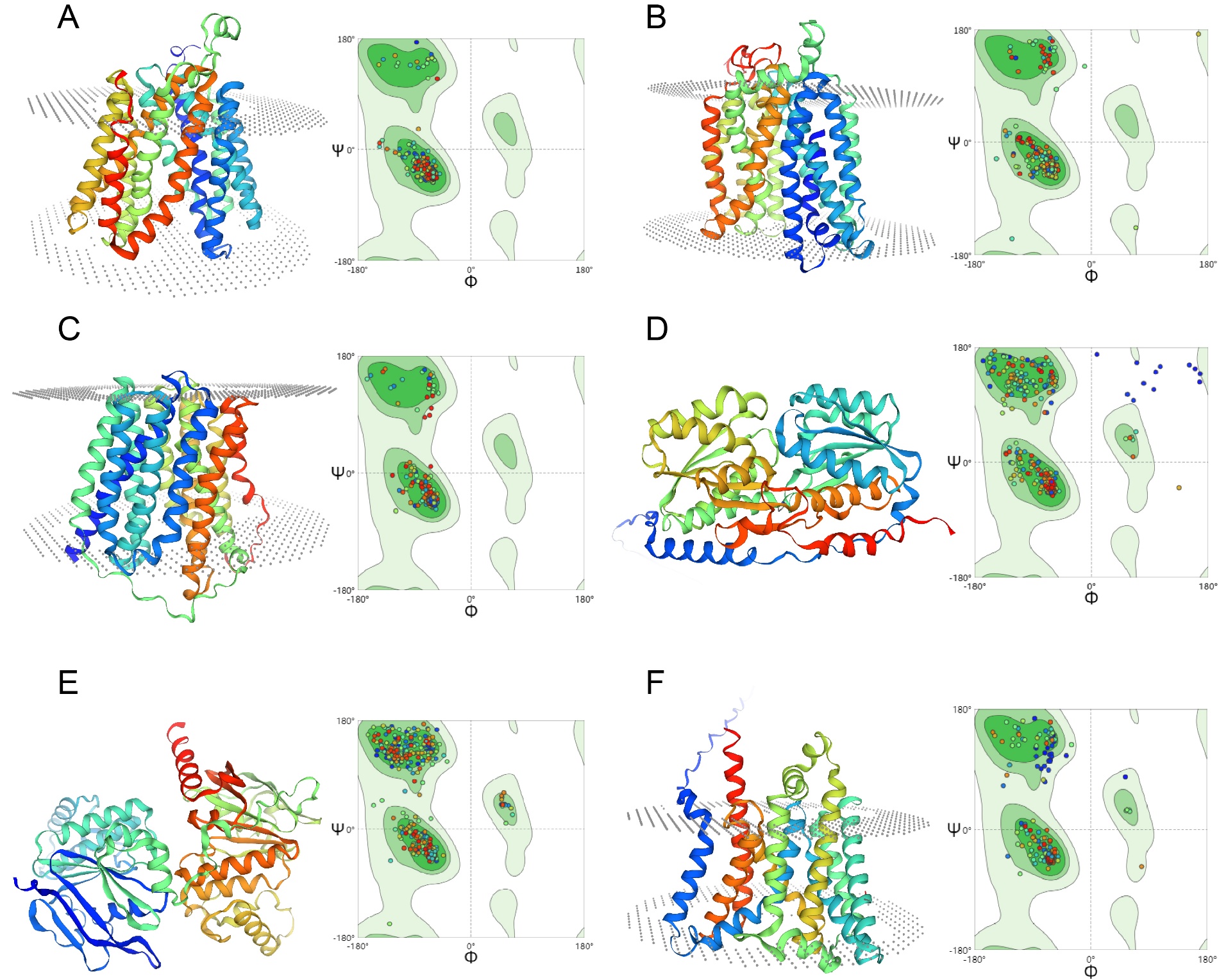


**Figure S8 3D structure and Ramachandran plot of four membrane proteins.** MFS transport proteins 0620 **(A)**,3572 **(B)**, 5299 **(C)** and ABC transport protein 4497 **(D)**, 4498 **(E)**, 4499 **(F)**.


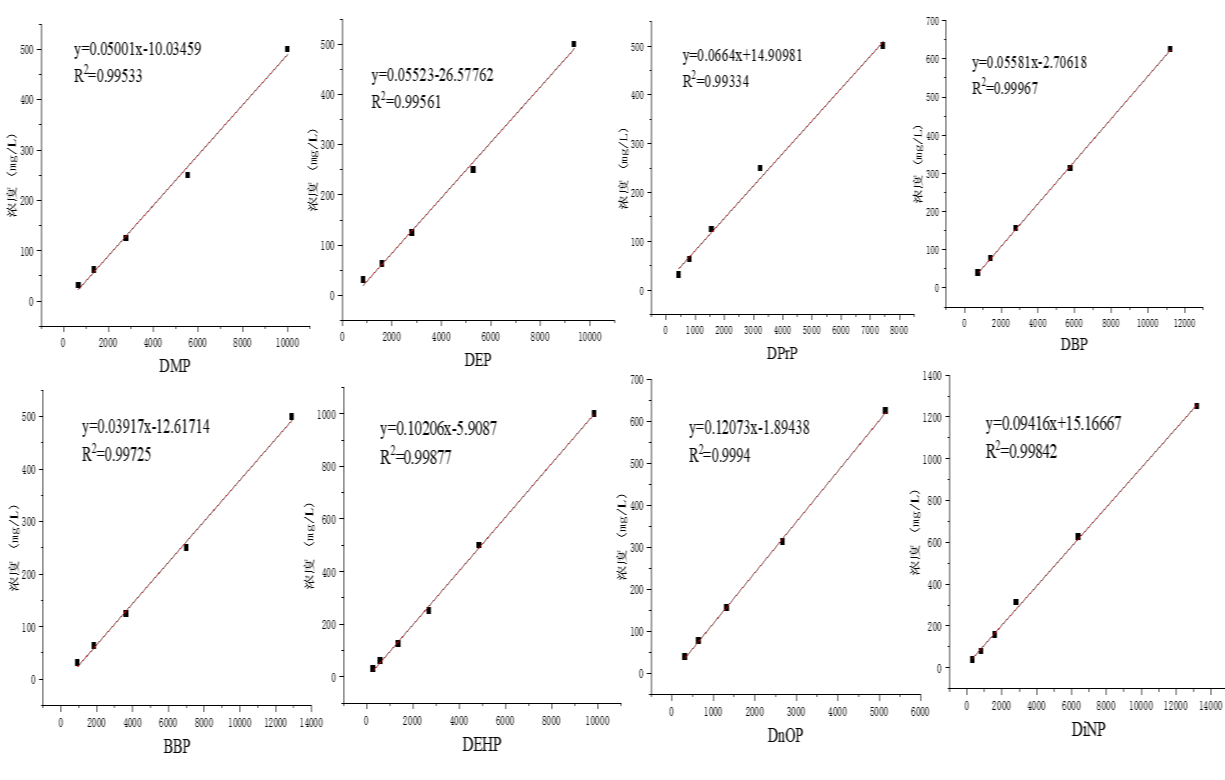


**Figure S9 Standard curve of 8 PAEs detected by HPLC**

Reference

1. Mori K, Niinuma K, Fujita M, Kamimura N, Masai E. 2018. DdvK, a Novel Major Facilitator Superfamily Transporter Essential for 5,5′-Dehydrodivanillate Uptake by *Sphingobium* Strain SYK-6. Applied and Environmental Microbiology 84:e01314-18.

2. Pernstich C, Senior L, MacInnes KA, Forsaith M, Curnow P. 2014. Expression, purification and reconstitution of the 4-hydroxybenzoate transporter PcaK from *Acinetobacter* sp. ADP1. Protein Expression and Purification 101:68-75.

3. Chaudhry MT, Huang Y, Shen XH, Poetsch A, Jiang CY, Liu SJ. 2007. Genome-wide investigation of aromatic acid transporters in *Corynebacterium glutamicum*. Microbiology-Sgm 153:857-865.

4. Mori K, Kamimura N, Masai E. 2018. Identification of the protocatechuate transporter gene in *Sphingobium* sp. strain SYK-6 and effects of overexpression on production of a value-added metabolite. Applied Microbiology and Biotechnology 102:4807-4816.

5. Wada A, T PÉ, Hirano R, Werner AZ, Kamimura N, Jacobson DA, Beckham GT, Masai E. 2021. Characterization of aromatic acid/proton symporters in *Pseudomonas putida* KT2440 toward efficient microbial conversion of lignin-related aromatics. Metabolic Engineering 64:167-179.

6. Choudhary A, Purohit H, Phale PS. 2017. Benzoate transport in *Pseudomonas putida* CSV86. Fems Microbiology Letters 364:fnx118.

7. Cillingová A, Zeman I, Tóth R, Nebohácová M, Duncková I, Hölcová M, Jakúbková M, Gérecová G, Pryszcz LP, Tomáska L, Gabaldón T, Gácser A, Nosek J. 2017. Eukaryotic transporters for hydroxyderivatives of benzoic acid. Scientific Reports 7:8998.

8. D'Arrigo I, Cardoso JGR, Rennig M, Sonnenschein N, Herrgård MJ, Long KS. 2019. Analysis of *Pseudomonas putida* growth on non-trivial carbon sources using transcriptomics and genome-scale modelling. Environmental Microbiology Reports 11:87-97.

9. Xu Y, Chen B, Chao H, Zhou NY. 2013. mhpT encodes an active transporter involved in 3-(3-hydroxyphenyl)propionate catabolism by *Escherichia coli* K-12. Applied and Environmental Microbiology 79:6362-6368.

10. Pardo I, Jha RK, Bermel RE, Bratti F, Gaddis M, McIntyre E, Michener W, Neidle EL, Dale T, Beckham GT, Johnson CW. 2020. Gene amplification, laboratory evolution, and biosensor screening reveal MucK as a terephthalic acid transporter in *Acinetobacter baylyi* ADP1. Metabolic Engineering 62:260-274.

11. Jiang D, Zhao Y, Wang X, Fan J, Heng J, Liu X, Feng W, Kang X, Huang B, Liu J, Zhang XC. 2013. Structure of the YajR transporter suggests a transport mechanism based on the conserved motif A. PNAS 110:14664-9.

12. Wu HH, Symersky J, Lu M. 2020. Structure and mechanism of a redesigned multidrug transporter from the Major Facilitator Superfamily. Scientific Reports 10:3949.

13. Liu TF, Li J, Qiu LQ, Zhang FM, Linhardt RJ, Zhong WH. 2020. Combined genomic and transcriptomic analysis of the dibutyl phthalate metabolic pathway in *Arthrobacter* sp. ZJUTW. Biotechnology and Bioengineering 117:3712-3726.

14. Hu T, Yang C, Hou ZY, Liu TF, Mei XT, Zheng LB, Zhong WH. 2022. Phthalate esters metabolic strain *Gordonia* sp. GZ-YC7, a potential soil degrader for high concentration di-(2-ethylhexyl) phthalate. Microorganisms 10:641-641.

15. Hou ZY, Pan HJ, Gu MJ, Chen XW, Ying TT, Qiao P, Cao JW, Wang HX, Hu T, Zheng LB, Zhong WH. 2024. Simultaneously degradation of various phthalate esters by *Rhodococcus* sp. AH-ZY2: Strain, omics and enzymatic study. Journal of Hazardous Materials 474:134776.
